# Plasma and serum metabolic analysis of healthy adults shows characteristic profiles by subjects’ sex and age

**DOI:** 10.1007/s11306-024-02108-z

**Published:** 2024-03-16

**Authors:** Rui Xu, Shiqi Zhang, Jieli Li, Jiangjiang Zhu

**Affiliations:** 1 Human Nutrition Program, Department of Human Sciences, The Ohio State University, Columbus, OH 43210 USA; 2 Comprehensive Cancer Center, The Ohio State University, Columbus, OH 43210 USA; 3 Department of Pathology, The Ohio State University, Columbus, OH 43210 USA

**Keywords:** Metabolomics, Pre-analytical factors, Blood processing methods, Sex, Age

## Abstract

**Introduction:**

Pre-analytical factors like sex, age, and blood processing methods introduce variability and bias, compromising data integrity, and thus deserve close attention.

**Objectives:**

This study aimed to explore the influence of participant characteristics (age and sex) and blood processing methods on the metabolic profile.

**Method:**

A Thermo UPLC-TSQ-Quantiva-QQQ Mass Spectrometer was used to analyze 175 metabolites across 9 classes in 208 paired serum and lithium heparin plasma samples from 51 females and 53 males.

**Results:**

Comparing paired serum and plasma samples from the same cohort, out of the 13 metabolites that showed significant changes, 4 compounds related to amino acids and derivatives had lower levels in plasma, and 5 other compounds had higher levels in plasma. Sex-based analysis revealed 12 significantly different metabolites, among which most amino acids and derivatives and nitrogen-containing compounds were higher in males, and other compounds were elevated in females. Interestingly, the volcano plot also confirms the similar patterns of amino acids and derivatives higher in males. The age-based analysis suggested that metabolites may undergo substantial alterations during the 25-35-year age range, indicating a potential metabolic turning point associated with the age group. Moreover, a more distinct difference between the 25–35 and above 35 age groups compared to the below 25 and 25–35 age groups was observed, with the most significant compound decreased in the above 35 age groups.

**Conclusion:**

These findings may contribute to the development of comprehensive metabolomics analyses with confounding factor-based adjustment and enhance the reliability and interpretability of future large-scale investigations.

**Supplementary Information:**

The online version contains supplementary material available at 10.1007/s11306-024-02108-z.

## Introduction

Metabolomics aims to achieve a comprehensive understanding of metabolites within biological systems. To maximize the reliability and significance of metabolomics analyses, it is increasingly recognized that larger sample sizes are beneficial. Including more samples offers several advantages, such as increased statistical power, better representation of biological variability, enhanced identification of biomarkers, improved reproducibility, and enhanced generalizability of findings (Tolstikov et al., 2020). Even though high-throughput technologies revolutionize metabolomics by making large-sample detection a reality (Plumb et al., 2023), however, performing large-scale sample processing in metabolomics can be challenging due to the introduction of pre-analytical factors. Pre-analytical factors refer to the various biological and pre-analytical variables that can impact the metabolite concentrations. These pre-analytical factors include factors during sample processing such as anticoagulant type, sample handling, sample processing, and storage procedures (Garwolińska et al., 2023), and the characteristics, such as sex and age(Cui et al., 2021). To surmount the barriers associated with sample collection and leverage the potential of high-throughput technologies, researchers often resort to adopting multiple testing (Navarro et al., 2023) and covariate adjustment (Bakusic et al., 2019), which are commonly employed to mitigate the influence of pre-analytical factors. Only when the pre-analytical factors are effectively avoided, the consistency and accuracy of test results and analysis process can be ensured, as well as reliable data generated. Therefore, the standardization of experimental protocols and the implementation of appropriate quality controls are necessarily needed.

While internal standards can address some inconsistencies in different sample sets (Yang et al., 2021), factors that cannot be normalized associated with blood processing method and participant characteristics remain challenging. The blood processing method used for analysis can also influence metabolite composition and levels (Stevens et al., 2019; Yin et al., 2013, 2015). Considering anticoagulants is crucial as different tissues or biofluids may have distinct metabolic profiles, affecting the interpretation of metabolomics data across studies. Sex differences can further impact hormonal, genetic, and physiological factors, leading to distinct metabolic patterns between males and females (Darst et al., 2019). Accounting for sex differences in a large-scale study allows for a better understanding of sex-specific metabolic patterns, potentially identifying sex-specific biomarkers or associations. In addition, metabolite levels and metabolic pathways can undergo substantial changes throughout the lifespan (Lassen et al., 2023). These age-related variations may be influenced by factors such as developmental stages, physiological changes, lifestyle, and environmental exposures (Darst et al., 2019). Considering age differences enhances the interpretation and generalizability of findings, especially when studying diseases or interventions that have age-specific implications.

In this study, we performed a targeted metabolomics analysis of 175 compounds from nine different classes on a set of 208 blood samples. Our primary objective was to examine the influence of three pre-analytical factors, including age and sex and anticoagulant (lithium heparin), on the metabolic profile of healthy individuals. By investigating these factors, we aim to provide valuable insights that can lay the foundation for more accurate results and robust interpretations across multiple datasets, ultimately advancing our understanding of metabolism and facilitating the integration of findings in future large-scale metabolomics investigations.

## Methods

### Materials

All the solvents used in this study, including LC/MS grade methanol, acetonitrile, water, ethanol, dimethyl sulfoxide, ammonium acetate, and acetic acid, were purchased from Fisher Scientific (Pittsburgh, PA, USA). Stable isotope labeled amino acid standards, 13 C/15 N-AminoAMix20 were purchased from Cambridge Isotope Laboratories (Tewksbury, MA, USA) to be used as internal standards for biological samples (Table S1). A total of 315 metabolite standards were purchased from MetaSci (Toronto, ON, Canada). Pooled human serum (used for quality control purposes) was purchased from Innovative Research (Novi, MI, USA). Detailed information regarding the standards is available in Table S2.

### Standard preparation

To prepare the metabolite standards for the study, each standard was dissolved in the most suitable solvent (water, ethanol, or dimethyl sulfoxide) based on their solubility. Stock solutions of 10 mM concentration were prepared for each standard. A gradient of standards ranging from 10 µM to 1 mM was generated by diluting the stock solutions. These gradients were used for direct infusion analysis. Additionally, mixes of each standard at a concentration of 10 µM were prepared to determine the retention times.

### Sample collection and preparation

A retrospective study was performed in the patient cohort. Residual serum or lithium heparin plasma samples from male or female patients were collected after overnight fasting from Dec 2021 to Apr 2022. All the samples that met the criteria sent to the Clinical Laboratory were aliquoted and stored at -80 °C, with the institutional review board approval at The Ohio State University Wexner Medical Center (IRB#2022C0138). Demographic information including age, race, and gender was obtained from the electronic medical records (EMR). Exclusion Criteria: 1. Cardiac vascular disease (including but not limited to Hypertension, Myocardial infarction, Valvular disease, Drug toxicity, Myocarditis, Takotsubo Cardiomyopathy, Cardiac hypertrophy, Arrhythmia, Chronic heart failure, Heart implantation, Cardiac contusion/trauma); Diabetes (type 1 and type 2); Crohn’s disease; Ulcerative colitis; Hyperlipidemia; Sepsis; Shock; Transgender; Thyroid disease; Cancer patients with chemotherapy; Pregnancy (female). To ensure diversity among our healthy control group, we made deliberate efforts to maintain gender balance, achieve a well-distributed age range (including a representation of elderly individuals), and secure participants who could provide both plasma and serum samples.

The plasma and serum samples underwent centrifugation (1500 g, 15 min, 4℃), followed by placement in cryogenic tubes, freezing in liquid nitrogen, and storage at − 80 ℃ during transportation. Subsequently, the samples were thawed at 4 °C and vortexed. A mixture comprising 50 µL of serum, 50 µL of spiking solution (consisting of 20 stable isotope-labeled amino acid standards, 13 C/15 N-AminoAMix20 in a 50% Water/50% Methanol solution), and 250 µL of HPLC-grade methanol was prepared and vortexed again. After storing the mixture at -20 °C for 20 min, it was subjected to centrifugation at 14,000 rpm for 20 min, and 150 µL of the supernatant was collected for subsequent analysis.

### Sample analysis

During the process of constructing the in-house standard library, each standard (from 10 μm to 1 mM) was injected directly into the mass spectrometer system. Parameters such as spray voltage, sheath gas, auxiliary gas, and sweep gas were optimized to achieve optimal precursor ion performance during the source optimization stage. Subsequently, product ion optimization was conducted using the RF lens for the precursor ion, with two product ions selected and a low mass exclusion set at 50 m/z. The scan type employed was the product scan. Both Q1 and Q3 resolutions were configured at 0.7 full width at half maximum (FWHM). The collision energy was set to 30 V, and the scan rate was maintained at 1000 Da/sec.

After collecting the individual parameters for the 315 metabolites, we adopted a pooling strategy. This involved combining every ten standards (each with non-overlapping molecular weights) at a concentration of 10 µM before conducting LC-MS analysis. The purpose of this method is to determine if each metabolite can be consistently detected using its previously optimized parameters within a mixed matrix. Additionally, this process allows for the recording of retention times. The mixtures of ten standards underwent chromatographic separation and were analyzed using the Thermo Vanquish UPLC system (ThermoFisher, Waltham, MA, USA) coupled with a TSQ Quantiva Triple Quadrupole Mass Spectrometer (ThermoFisher). The mass spectrometer was equipped with a heated electrospray ionization probe from Thermo Fisher (MA, USA). The chromatographic separation was conducted on an XBridge BEH Amide XP column (130Å, 2.5 μm, 2.1 mm X 150 mm, 2.5 μm particle size) manufactured by Waters Corporation (Milford, MA). The analysis employed the Multiple Reaction Monitoring (MRM) scan type, utilizing a Selected Reaction Monitoring (SRM) table that contained the optimized production ions obtained in the previous step. For the chromatographic analysis, mobile phase A comprised a mixture of 5 mM ammonium acetate in acetonitrile /water (10:90, v/v) with 0.1% acetic acid, while mobile phase B was composed of 5 mM ammonium acetate in acetonitrile /water (90:10, v/v) with 0.1% acetic acid. A linear gradient elution program was implemented, initiating with 70% B and gradually decreasing to 30% B within 5 min. Subsequently, the mobile-phase composition was sustained at 30% B for 4 min, after which it was reverted to 70% B within 2 min and maintained for an additional 2 min. The entire run duration lasted 13 min. The flow rate was set to 0.3 mL/min, and the column temperature was maintained at 40 °C.

Through manual inspection of peaks in Xcalibur Quanbrowser (ThermoFisher), the retention time and retention time windows were extracted and incorporated into the SRM table to optimize the targeted method. Overall, 315 metabolites with high-quality signals from standard tests were added to our SRM tables. Subsequently, all prepared plasma and serum samples were analyzed using the target method for robust and reproducible results. To assess instrument stability during testing and enable data normalization, commercial human serum quality control (QC) and blank control samples (mobile phase solution) were analyzed following every ten injections of biological samples. Initially, 107 participants were included, each providing both serum and plasma samples. Subsequently, 6 plasma samples were excluded as outliers, and 208 samples were kept for subsequent analysis.

### Data preprocessing

Following manual peak-picking and peak-correction using Xcalibur Quanbrowser (ThermoFisher), a data matrix was generated, encompassing the peak intensity of all metabolites across all samples. Metabolites with intensity < 10,000 arbitrary units (a.u.), on average were removed to avoid potential interference from noise signal. This data matrix served as the foundation for subsequent analytical processes and further analysis. To facilitate comprehensive analysis beyond individual metabolite levels, all the identified metabolites were classified into 9 distinct classes based on the Human Metabolome Database (HMDB) (Wishart et al., 2018). Namely, the classes used entailed: amino acids and derivatives, aromatic acids and derivatives, carbohydrates and derivatives, carboxylic acids and derivatives, lipids, nitrogen-containing compounds, nucleic acid-related compounds, Vitamins and coenzymes, and others. This classification enabled the examination and interpretation of data not only at the metabolite level but also at the broader class level.

### Statistical analysis

Overall, the independence test among 3 factors was performed by covariate analysis with a p-value indicating if the 3 factors were regressed to each other regarding each metabolite. First, univariate analyses were conducted to assess the relationship between metabolites and the variables of interest. Logistic regression analyses were employed to examine the association between each metabolite and sex or blood processing methods, while linear regression analyses were performed to investigate the relationship between each metabolite and age. To adjust for multiple comparisons, the FDR correction was utilized, establishing a significance threshold of *p* < 0.05. Metabolites that demonstrated statistical significance after applying the FDR correction (*p* < 0.05) were considered to significantly fit the models, indicating their good regression ability of the models. Subsequently, to establish a predetermined threshold (|log2(foldchange)| > 0.3) for fold change and incorporate a rigorous significance test, volcano plots were employed to assess the variations in metabolite levels across different blood processing methods and sexes. For age analysis, the metabolite intensity of each metabolite or metabolite class at specific age or age intervals is averaged. A line chart was employed to visually depict the overall temporal pattern of these aggregated values, and showcase the changing trend across the different age or age groups. A pairwise t-test was performed for the significance test during age analysis. Lastly, to comprehensively elucidate the contribution of the identified metabolites towards classification, Partial Least Squares Discriminant Analysis (PLS-DA) was employed to integrate metabolites and classify individuals based on their sex, blood processing methods, and age groups (< 25, 25–35, and > 35). Metabolites exhibiting a Variable Importance in Projection (VIP) score > 1.5 were considered significant and subsequently selected in PLS-DA models. The significant metabolites obtained from the regression analysis, volcano plot, and PLS-DA were combined and visualized using a Venn plot. This plot showed how the changes in metabolite levels varied across the different analysis models. Additionally, the selected metabolites were grouped into classes and represented in a pie chart, indicating which classes were most affected by these pre-analytical factors.

## Results

**Fig. 1:**
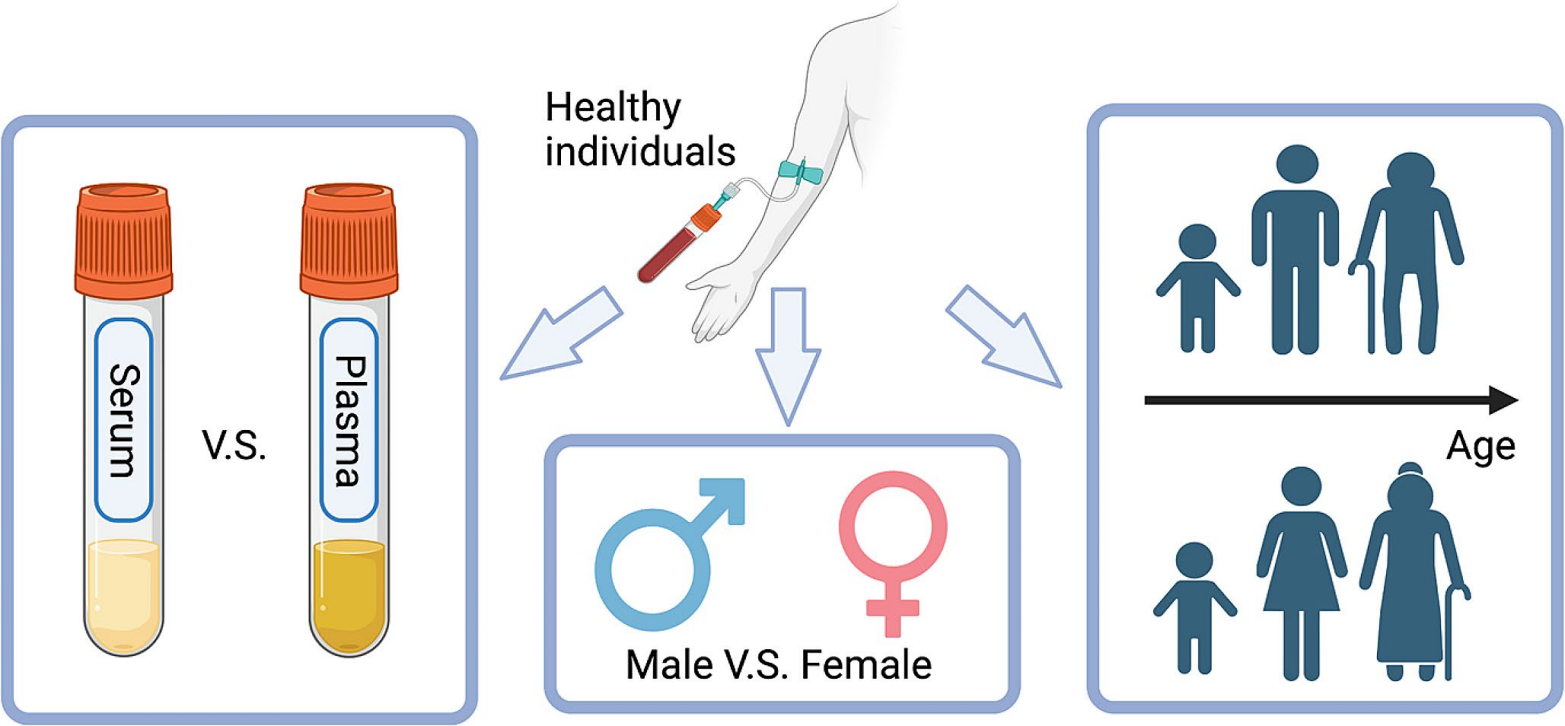
A. The schematic workflow of the study

To investigate the influence of three common pre-analytical factors (blood processing methods, sex, and age) on the metabolic profile of healthy individuals, 208 samples were assembled from a group of healthy individuals (Fig. 1). The samples were selected to ensure an even distribution across blood processing methods, encompassing both sexes and covering a specific age range. The details of the sample group are provided in Table 1. To assess the relationship between these factors, independence tests were conducted. The independence tests aimed to determine whether there were any associations or dependencies between any two factors concerning all the detected metabolites. The results revealed that all factors were found to be independent of each other (p-value > 0.05) concerning all the measured metabolites. This suggests that the variations observed in the metabolic profiles can be attributed to each specific factor independently, rather than being influenced by interdependencies among the factors. Thus, each factor was analyzed separately. After QC check, 175 metabolites with CV < 0.3 were recognized as reliable identification (Figure S1, Table S3). Figures S2A and S2B illustrate the distribution of metabolites across different classes within the target list and the subset of metabolites detected on the Quantiva instrument. As shown in Figures S2A and S2B, our method aimed to scan for 315 metabolites and reliably detected 175 metabolites, our detection covers major metabolite classes such as amino acids and derivatives, carboxylic acids and derivatives, and aromatic acids and derivatives, which were frequently reported in disease biomarkers studies (Gold et al., 2022), therefore, we believe our evaluation method is unbiased and will reflect diverse differences of metabolites across different classes.

**Table 1:** Characteristics summary of the study population

Biospecimens	Plasma (*n* = 101)		Serum (*n* = 107)	
Sex	Female (*n* = 48)	Male (*n* = 53)	Female (*n* = 54)	Male (*n* = 53)
Age				
< 25	14	11	16	11
25–35	28	26	32	26
35–45	2	12	2	12
45–55		4		4
55–65	3		3	
65–75	1		1	

**Fig. 2:**
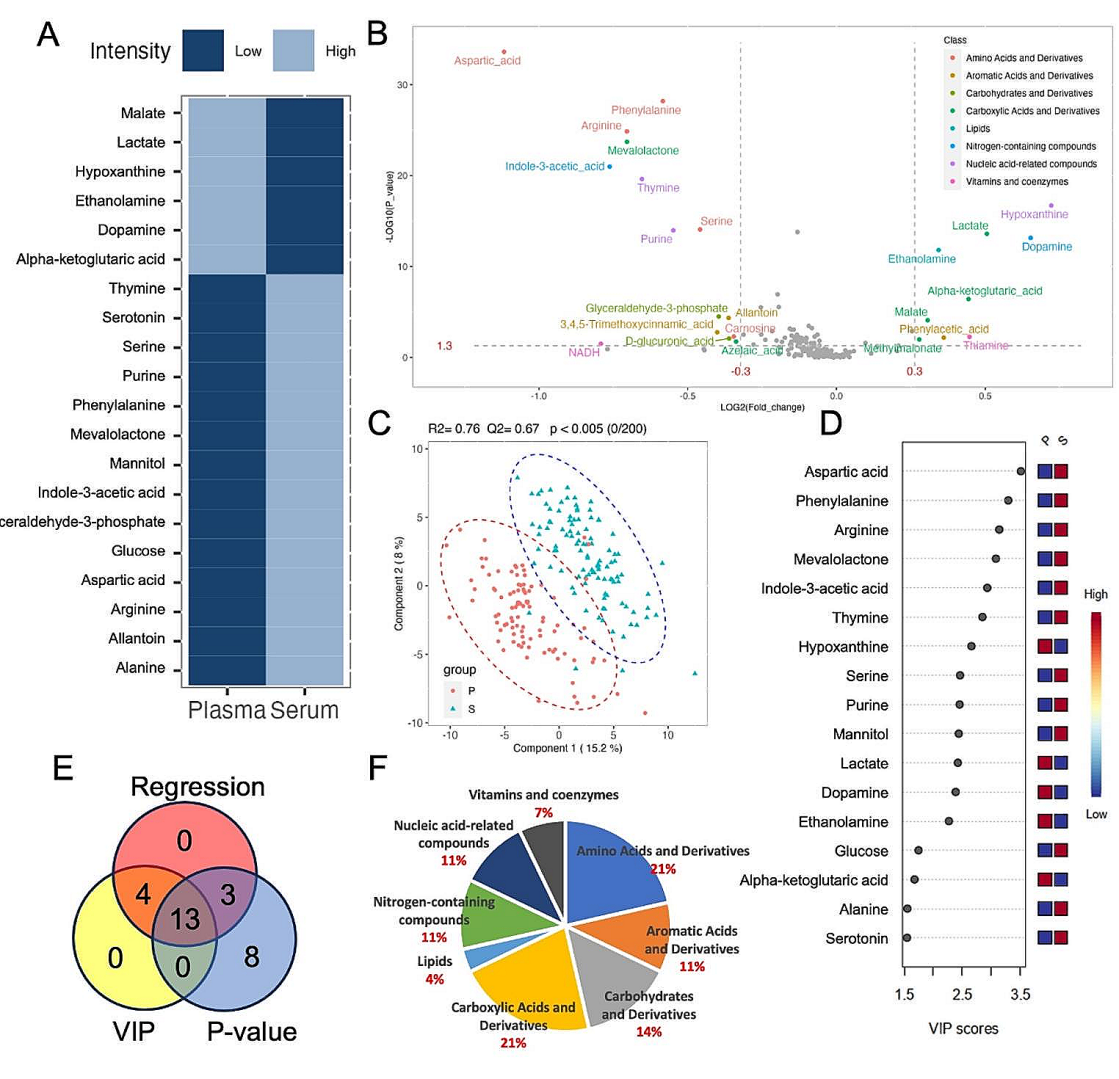
(**A**) Color map illustrating metabolites exhibiting significant differences between plasma and serum samples from the same group of individuals, selected using logistic regression analysis with FDR correction (*p* < 0.05); (**B**) Volcano plot depicting the selection of differential metabolites between serum and plasma based on fold change and p-value; the color of each dot corresponds to the respective metabolite class. (**C**) PLS-DA plot illustrating the discrimination between plasma (P) and serum (S) samples based on their metabolic profiles; (**D**) VIP plot presenting the top-ranked metabolites, identified with VIP scores greater than 1.5, derived from the PLS-DA plot in Fig. 2C; (**E**) Venn plot displaying the shared and unique sets of differential metabolites selected through above regression analysis, volcano plot, and PLS-DA; (**F**) Distribution of selected metabolites from the analysis conducted above across compound classes

To find the robust differential metabolites between plasma and serum samples, we employed three different statistical analysis approaches. Figure 2A illustrates a color map depicting the results of logistic regression. The color map highlights 20 metabolites that exhibited a significant fit to the logistic regression model at a significance level of 0.05 after applying the FDR correction. Among these metabolites, 14 were found to be significantly higher in serum compared to plasma. In Fig. 2B, the volcano plot displays 24 metabolites that exhibit significant changes. Out of these metabolites, 15 were found to be higher in serum. The colors of the dots in the volcano plot represent their respective compound classes. Notably, amino acids and derivatives highlighted in the volcano plot were found to be significantly higher in serum, whereas carboxylic acids and derivatives were significantly higher in plasma. In the PLS-DA scoring plot (Fig. 2C), a discernible separation between plasma and serum is observed based on their metabolic profiles. The PLS-DA analysis reveals that Component 1 accounts for 15.1% of the variation, while Component 2 accounts for 8% of the variation. The R^2^ value of 0.76 and Q^2^ value of 0.67 indicate a good fit and predictive ability of the PLS-DA model. Additionally, the permutation test (*n* = 200) yielded a p-value of less than 0.005, confirming the statistical significance of the observed separation between plasma and serum based on their metabolic profiles. In the VIP score plot (Fig. 2D), it is evident that the top-ranked metabolites exhibit significantly higher intensity levels in serum. Additionally, upon summarizing all significant metabolites mentioned earlier, a total of 13 metabolites in 5 classes are considered to be the most responsive to blood sample type difference (Fig. 2E; Table 2). Alpha-ketoglutaric acid, lactate, ethanolamine, dopamine, and hypoxanthine were higher in the plasma while others were higher in the serum. This consistent pattern reinforces the significant metabolic differences observed between serum and plasma, irrespective of the statistical analysis tools used. In Fig. 2F, the classification of 28 selected metabolites in Fig. 2E is presented. Notably, amino acids and derivatives (21%) and carboxylic acids and derivatives (21%) were the compound classes most influenced by the blood processing methods.

**Table 2:** Common significant differential metabolites between plasma and serum generated from 3 statistical models

Compound name	Compound class	VIP	Regression significance (p-value after FDR correction)	Foldchange (Plasma/Serum)	Student t-test (p-value)
Arginine	Amino Acids and Derivatives	3.14	2.40E-22	0.61	1.35E-25
Aspartic acid		3.51	7.85E-26	0.46	2.37E-34
Phenylalanine		3.29	4.26E-23	0.67	6.46E-29
Serine		2.46	4.25E-11	0.64	2.42E-20
Alpha-ketoglutaric acid	Carboxylic Acids and Derivatives	1.68	1.33E-02	1.36	3.78E-07
Lactate		2.42	2.57E-11	1.42	2.50E-14
Mevalolactone		3.08	3.92E-22	0.61	1.90E-24
Ethanolamine	Lipids	2.27	2.86E-09	1.27	1.54E-12
Dopamine	Nitrogen-containing compounds	2.39	7.33E-09	1.57	7.10E-14
Indole-3-aceticacid		2.93	1.49E-16	0.59	1.00E-21
Hypoxanthine	Nucleic acid-related compounds	2.66	1.16E-10	1.65	1.94E-17
Purine		2.45	1.06E-12	0.73	8.39E-15
Thymine		2.85	4.71E-17	0.76	1.70E-03

**Fig. 3:**
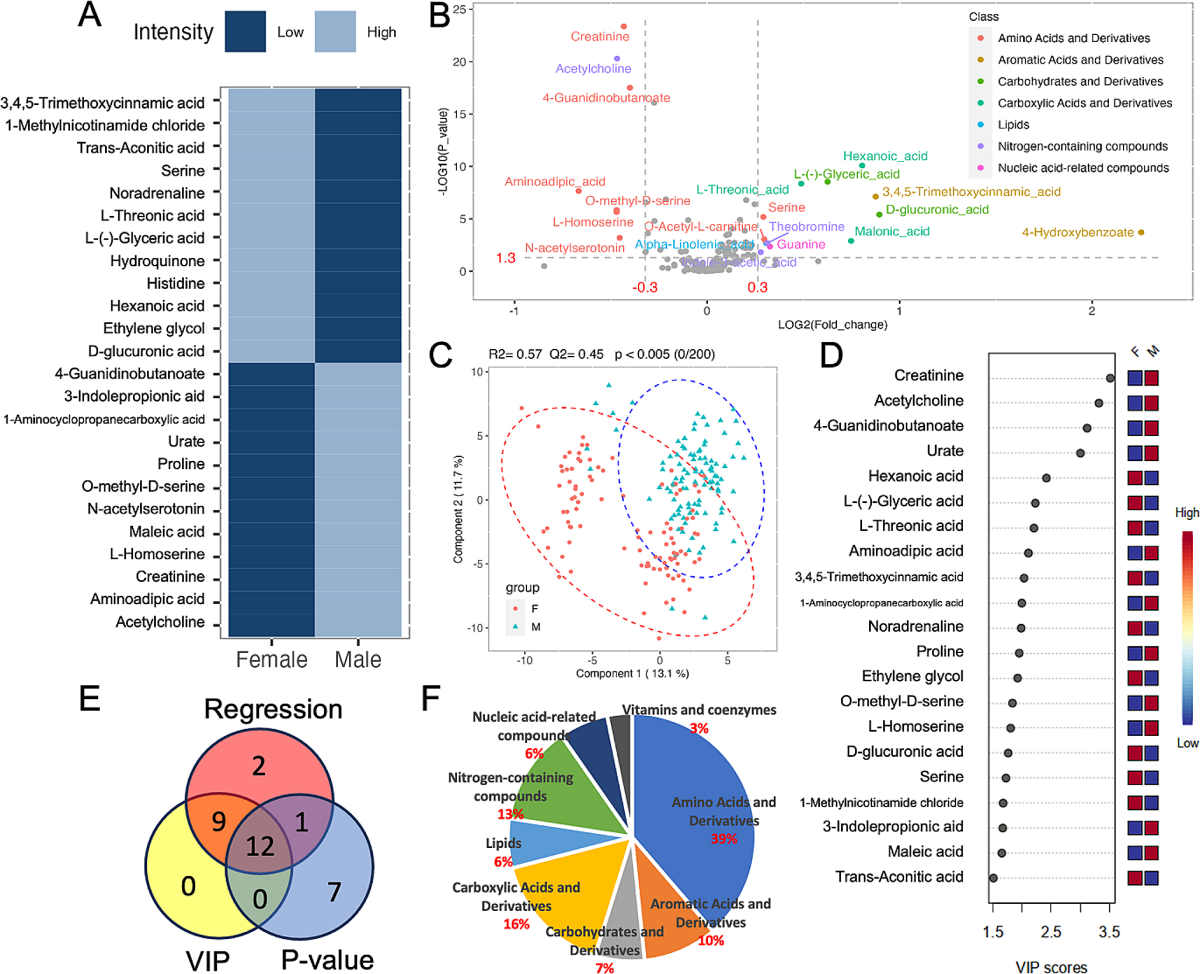
(**A**) Color map illustrating metabolites exhibiting significant differences between males and females, selected using logistic regression analysis with FDR correction (*p* < 0.05); (**B**) Volcano plot depicting the selection of differential metabolites between males and females based on fold change and p-value; the color of each dot corresponds to the respective metabolite class. (**C**) PLS-DA plot illustrating the discrimination between male (M) and female (F) based on their metabolic profiles; (**D**) VIP plot presenting the top-ranked metabolites, identified with VIP scores greater than 1.5, derived from the PLS-DA plot in Fig. 2C; (**E**) Venn plot displaying the shared and unique sets of differential metabolites selected through above regression analysis, volcano plot, and PLS-DA; (**F**) Distribution of selected metabolites from the analysis conducted above across compound classes

Similar to blood processing methods, we did three types of statistical analyses to reveal the sex-dependent metabolite differences. Logistic regression analysis revealed that in Figs. 3A and 24 metabolites, such as 3,4,5-trimethoxycinnamic acid, 1-methylnicotinamide chloride, and trans-aconitic acid, showed significant differences between females and males. Among these metabolites, the first half were detected at higher levels in females, while the second half exhibited higher levels in males. The volcano plot in Fig. 3B highlighted 21 metabolites that satisfied the criteria for both |Log2(foldchange)| >0.3 and p-value < 0.05, thus indicating their significance. The color of the dots in the plot indicated that the highlighted amino acids and derivatives were significantly lower in females, whereas aromatic acids and derivatives, carbohydrates and derivatives, and carboxylic acids and derivatives were higher in males. Examining the PLS-DA plot in Fig. 3C, despite the presence of some outliers beyond the 95% confidence interval among males, a certain level of separation between female and male samples was still observed. This separation was confirmed by the permutation test (*n* = 200) with a p-value < 0.005. The corresponding top VIP list (Fig. 3D) comprised a total of 20 metabolites that significantly contributed to the separation in the PLS-DA score plot. Additionally, the Venn plot (Fig. 3E) demonstrated that across the three statistical methods, 12 metabolites in 5 classes were consistently identified as significantly different by sex (Table 3), in which amino acids and derivatives were dominantly influenced. Among all the selected metabolites, it was observed that not only amino acids and derivatives, but also carboxylic acids and derivatives were influenced by sex. These findings suggest that these particular classes of metabolites warrant greater consideration when addressing cases where sex-related influences of metabolomics data may be at play.

**Table 3:** Common significant differential metabolites between female and male samples generated from 3 statistical models

Compound name	Compound class	VIP	Regression significance (p-value after FDR correction)	Foldchange (Female/ Male)	Student t-test (p-value)
4-Guanidinobutanoate	Amino Acids and Derivatives	3.12	1.26E-15	0.76	3.07E-18
Aminoadipic acid		2.11	3.72E-05	0.63	2.24E-08
Creatinine		3.52	1.06E-20	0.74	4.36E-24
L-Homoserine		1.8	9.84E-05	0.72	2.24E-06
O-methyl-D-serine		1.83	8.44E-05	0.72	1.44E-06
Serine		1.72	9.36E-04	1.22	6.49E-06
3,4,5-Trimethoxycinnamic acid	Aromatic Acids and Derivatives	2.03	2.15E-07	1.83	7.45E-08
D-glucuronic acid	Carbohydrates and Derivatives	1.76	9.04E-07	1.86	3.91E-06
L-(-)-Glyceric acid		2.23	1.17E-06	1.54	2.89E-09
Hexanoic acid	Carboxylic Acids and Derivatives	1.92	1.42E-08	1.75	8.16E-11
L-Threonic acid		2.21	8.94E-07	1.4	4.38E-09
Acetylcholine	Nitrogen-containing compounds	3.32	5.21E-18	0.72	5.08E-21

**Fig. 4:**
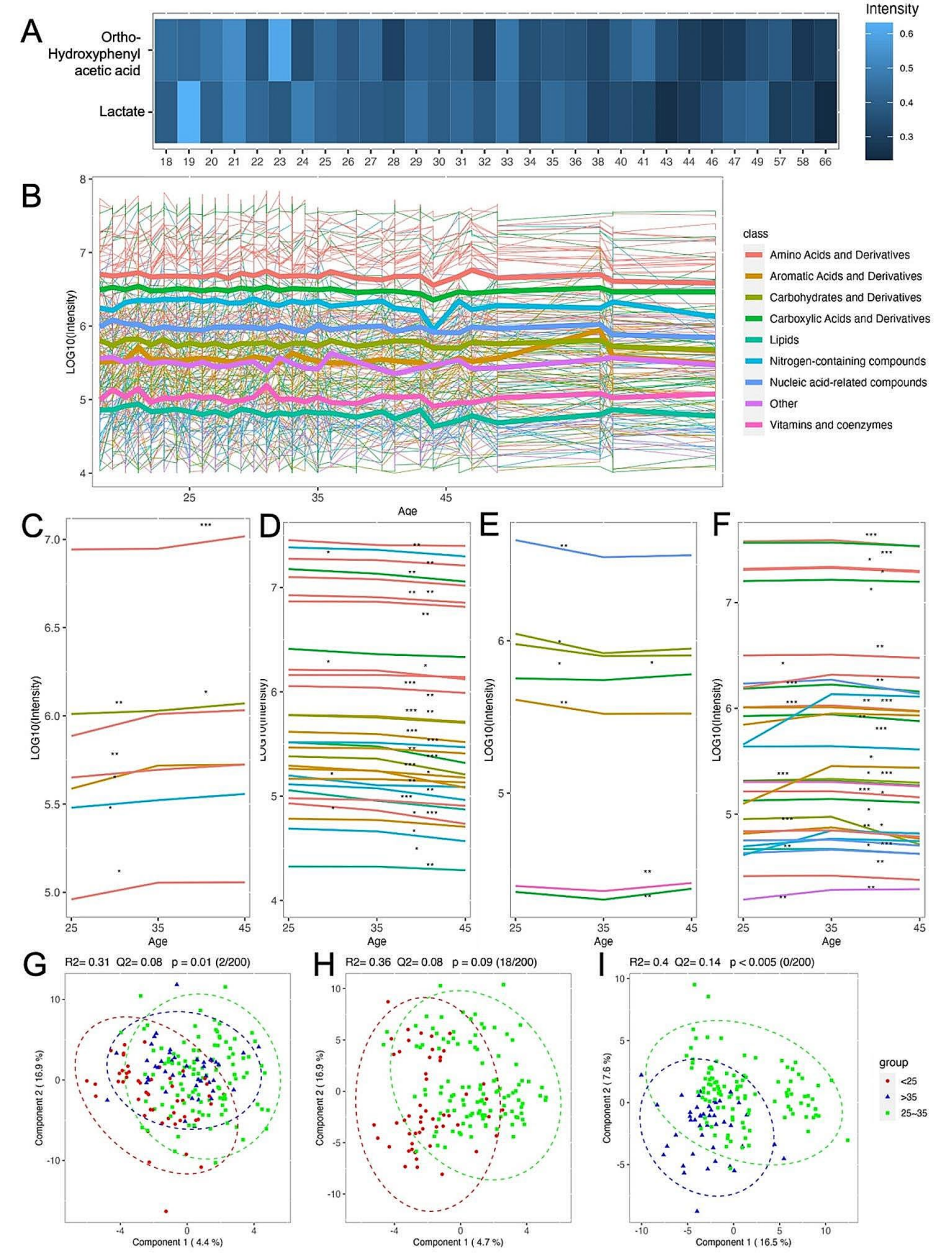
(**A**) Color map illustrating metabolites exhibiting significant changing trend by age, selected using liner regression analysis with FDR correction (*p* < 0.05); (**B**) Line chart depicting the overall temporal pattern of aggregated metabolite/class values, showcasing the changing trend across different age groups; the color of the lines represents the respective metabolite classes; C, D, E, F. The significant continuous increasing metabolites (**C**), increasing and then decreasing metabolites (D), decreasing and then increasing metabolites (**E**), decreasing metabolites (**F**) across age groups (< 25, 25–35, > 35 years); G, H, I. PLS-DA plot illustrating the discrimination among three age groups (**G**); between < 25 and 25 ~ 35 years (**H**); and between 25 ~ 35 and > 35 years (**H**)

When considering age analysis, the approach differs due to the nature of age being a continuous variable. Therefore, a linear regression analysis was performed to explore the relationship between each metabolite and the age of the blood donor. Following FDR correction on p-value to account for regression significance, only two metabolites were found to exhibit a significant linear correlation with age (Fig. 4A). Interestingly, both metabolites, namely ortho-hydroxyphenylacetic acid, and lactate, demonstrated an inverse relationship with patient age as they were found to be decreasing as age increased.

To investigate whether specific classes of metabolites also exhibited a similar decreasing pattern with age, a line chart was used to visualize the changes in individual metabolites as well as the aggregated values for each metabolite class across different age ranges (Fig. 4B). However, no discernible pattern emerged from this analysis. To simplify the analyses, the continuous age variable was divided into three age groups: 25 ± 5 years, 35 ± 5 years, and 45 ± 5 years. Furthermore, metabolites that exhibited significant changes among these three age groups, as defined by the pairwise t-test, were further categorized into four-line charts based on their changing trends. Figure 4C, D and E, and 4F present the metabolites that demonstrated continuous increase, continuous decrease, decrease followed by an increase, and increase followed by a decrease trends, respectively. The analysis reveals that a much larger number of metabolites experienced a decrease with age (Fig. 4D, *n* = 23) compared to those that exhibited an increase (Fig. 4C, *n* = 1). Additionally, a notable observation is that a greater number of metabolites displayed an increasing trend followed by a subsequent decrease (Fig. 4F, *n* = 18) as opposed to a decreasing trend followed by an increase (Fig. 4E, *n* = 3). Moreover, the number of decrease lines as well as significance markers indicates that a more pronounced and statistically significant decrease occurs between age groups 35 ± 5 and 45 ± 5 years. These significantly changed metabolites corresponding to Fig. 4C-F are listed in Table S4 and more details about the statistics are included in Table S5. To comprehensively illustrate the integrated metabolites’ influence on these three age groups, a PLS-DA plot encompassing all three groups was generated (Fig. 4G). Surprisingly, the plot revealed an overall trend in the metabolic profile characterized by a distinct shift occurring between the ages of 25 and 35. Specifically, individuals below 25 years of age exhibited the greatest separation from those in the 25–35 age range, and individuals above 35 years displayed a return toward the metabolic profile observed in individuals below 25 years. This finding suggests that, in comparison to continuous changes, metabolites may undergo more substantial alterations during the 25-35-year period through a turning point mechanism. Furthermore, the pairwise PLS-DA analysis demonstrated that the separation between the 25–35 and above 35 age groups was more pronounced than that between the below 25 and 25–35 age groups. Remarkably, these findings align with the results observed in the previous line charts. This observation was supported by multiple indicators, including component 1 (accounting for 16.5% of the variation), R2 (0.4), Q2 (0.14), and a permutation test p-value (< 0.005). These results collectively emphasize the significance of the age range from 25 to 35 years in driving notable changes in the metabolic profile, indicating a distinct metabolic shift during this period.

## Discussion

With advancements in analytical and computational techniques, the realization of conducting extensive high-throughput and high-dimensional data analysis in metabolomics research has become increasingly feasible. To overcome the bias introduced by some pre-analytical factors, such as age, sex, and blood processing methods, it is crucial to gain a better understanding of how these factors impact the measured metabolites. In this study, we first compared the metabolic profiles of serum and plasma samples with a matched sample set from 104 healthy individuals. Sample type-based metabolomics analyses have been the focus of multiple studies (Kaluarachchi et al., 2018; Liu et al., 2010; Nishiumi et al., 2018; Teahan et al., 2006; Yu et al., 2011). These studies have consistently reported significant differences in polar metabolites and lipids between the two blood processing methods, that is, plasma and serum, and they have attributed the blood processing approaches as a contributing factor to the divergent metabolome.

Furthermore, the means of sample processing can also contribute to the wide array of findings in the literature (Ishikawa et al., 2014). collected EDTA plasma, with a known anticoagulant used in blood processing. The addition of anticoagulants in blood collection tubes, which can chelate the calcium in the blood, has been shown to influence the metabolome and lipidome of collected plasma samples. Other agents, such as acid citrate dextrose or citrate, can also contribute to the overall amino acid profile of biological samples when analyzed with high throughput -omics technologies (Sotelo-Orozco et al., 2021a).

Previous investigations have consistently reported a significantly higher abundance of amino acids (such as phenylalanine, arginine, and serine) in serum samples (Liu et al., 2010; Yu et al., 2011), which aligns with the outcomes of our study. However, other amino acids such as serine, glycine, proline, and isoleucine were detected with significance in previous studies but were not found to be significantly different in our study. Furthermore, serum samples have been reported to exhibit higher levels of glucose, a vital energy source, in accordance with previous research findings (Ladenson et al., 1974; Liu et al., 2010). Lactate, a byproduct of glucose metabolism, displayed significantly lower concentrations in serum samples in our analysis. This observation in our study could potentially be attributed to increased glucose utilization during incubation, wherein biochemical metabolism occurred in plasma samples. Finally, our findings revealed a discernible pattern wherein a greater number of metabolites exhibited significantly higher levels in serum samples compared to plasma samples. This observation can be partially attributed to the removal of protein in the serum samples due to coagulation (Kronenberg et al., 1998). During serum preparation, the volume occupied by the proteins was removed, and the remaining constituents with lower molecular weights were distributed in a smaller volume. As a result, these constituents become more concentrated in the serum samples.

Previous research has reported sex- and age-related disparities in blood metabolites (Chaleckis et al. 2016; Ishikawa et al., 2014; Saito et al., 2016). Concerning females, males tend to utilize more energy to maintain cellular needs accompanied by additional muscle (Sotelo-Orozco et al., 2021b). As skeletal muscle density is reduced in aging adults, this contributes to metabolic deregulations in both genders as well (Palmer et al., 2022). Cellular metabolism also relies on the orchestrated action of specific enzymatic products derived from gene expression rendering genetic variation a contributing factor to diverse metabolic perturbations (Carthew, 2021). Thus, it is no surprise that energy metabolism varies within a diverse population (de Boer et al., 1988). Another study has also revealed significant gender disparities not only in amino acid metabolism but also in lipid metabolism (Krumsiek et al., 2015). Nevertheless, our study differs from these prior investigations in terms of the significant metabolites identified. This discrepancy can be attributed to the different focuses, which centers on lipidomics (Ishikawa et al., 2014), and the other centers on the Japanese population(Saito et al., 2016). As a result, there is no overlap in the significant metabolites identified between these previous studies and our current investigation. Nevertheless, certain detected metabolites, such as noradrenaline, a stress hormone and neurotransmitter in the central and peripheral nervous systems, and creatinine, a byproduct of muscle metabolism, are expected to exhibit sex differences, and we observed a significantly higher level of noradrenaline in females in our study. This is thought to be a result of estrogen’s ability to block reuptake of noradrenaline (Sudhir et al., 1996). However, it is important to note that the relationship between noradrenaline levels and sex lacks consistent conclusions, as noradrenaline levels can be influenced by diverse factors, including genetics and environmental factors such as stress levels and diet (Pickering, 1997; Welberg et al., 2001). Similarly, in the case of creatinine, a waste product produced by muscle metabolism, the observed sex differences are mainly associated with disparities in muscle mass, and we detected a significant elevation in creatinine levels among males compared to females.

Regarding age-associated differences, we observed limited changes among the metabolites we detected, with ortho-hydroxyphenylacetic acid and lactate being the notable exceptions, both exhibiting a decrease in older participants. This outcome may be attributed to the relatively concentrated age distribution of our population, with a majority (92.3%) falling below the age of 45 years. Previous studies examining age-related disparities in blood metabolites have also reported significant results(Chaleckis et al., 2016; Ishikawa et al., 2014; Saito et al., 2014, 2016), likely due to the broader age ranges selected, such as the inclusion of younger populations (25–34 years old) and older populations (55–64 years old), which differ significantly from the age groups represented in our sample. In comparison to specimen and sex analysis, histidine emerges as one of several metabolites concurrently influenced by age and sex, aligning with findings from another study (Dunn et al., 2015). However, our research uniquely demonstrates that age and sex act as independent influencing factors. Overall, it is noteworthy that in one study, age-associated changes were more prominent than those associated with differences in sex or race within the population group. Similarly, our study exhibits a similar trend in terms of the quantity of differential metabolites (Lawton et al., 2008). It must be acknowledged that with age, there are numerous factors influencing metabolism. For instance, both the composition of gut microbiota and dietary patterns significantly impact long-term metabolism. Human gut microbiota is established during infancy, responds to environmental exposures during childhood, and gradually matures. Subsequently, it remains relatively stable until diversity declines in old age. The gut microbiota’s association with metabolites encompasses various mechanisms, such as the generation of amino acid metabolites and the regulation of energy metabolism (Fan et al., 2021). Complex nutritional sensing pathways intricately fine-tune metabolic responses to dietary amino acids in a highly conserved manner (Soultoukis et al., 2016). In turn, these metabolic responses influence long-term human health. Due to limited data, these hypotheses may need to be explored in future research.

Overall, our study used multiple statistical methods to identify the differential metabolites influenced by sex, age, and blood processing methods, providing a more robust and reliable exploring result and reference for future studies. We also have to admit that more characteristic information would be needed for additional biological discussion, such as diet patterns and race, and we will consider these factors in our future study design.

## Conclusion

In this study, we found significant differences in metabolite profiles between two blood processing methods, serum and plasma. Several representative amino acids such as arginine aspartic acid, phenylalanine, and serine found to be significantly higher in serum samples, which is consistent with previous literature. Not surprisingly, we also observed sex-based metabolic sex differences, such as higher noradrenaline levels in females and higher creatinine levels in males. Age-related metabolic differences were tracked in our study as well, but limited conclusions can be drawn. However, our results did show a decreasing trend in certain metabolites (e.g., ortho-hydroxyphenylacetic acid and lactate) within older participants concerning those who are young. These findings can contribute to the understanding of metabolite variations across blood processing methods and participant characteristics, emphasizing the importance of considering these factors in metabolomics studies.

### Electronic supplementary material

Below is the link to the electronic supplementary material.

Supplementary Material 1
