# Supplementary material for: Plasma and serum metabolic analysis of healthy adults shows characteristic profiles by subjects’ sex and age
Source: Metabolomics. 2024 Mar 16;20(2):43. doi: 10.1007/s11306-024-02108-z (PMC10943143; doi:10.1007/s11306-024-02108-z)
Supplement: Supplementary file 1 — Supplementary Material 1 [file 11306_2024_2108_MOESM1_ESM.docx]

*Supplementary information for*

**Plasma and serum metabolic analysis of healthy adults shows characteristic profiles by subjects' sex and age**

Rui Xu^1, 2^, Shiqi Zhang^1, 2^, *Jieli Li^3^, *Jiangjiang Zhu ^1,2^

**Author affiliations:**

1. Human Nutrition Program, Department of Human Sciences, The Ohio State University, Columbus, Ohio 43210

2. Comprehensive Cancer Center, The Ohio State University, Columbus, Ohio 43210

3. Department of Pathology, The Ohio State University, Columbus, OH 43210, USA

*Co-corresponding:

Jieli Li, M.D., Ph.D.

Tel: 614-685-0057, Email: jieli.li@osumc.edu

Jiangjiang Zhu, Ph.D.,

Tel: 614-685-2226, Email: [zhu.2484@osu.edu](mailto:zhu.2484@osu.edu)

**Figure S1. The CV distribution of 315 metabolites based on the analyses of QC samples.**

**Figure S2.** A. Distribution of metabolites in the detection method across compound classes; B. Distribution of metabolites detected across compound classes

***
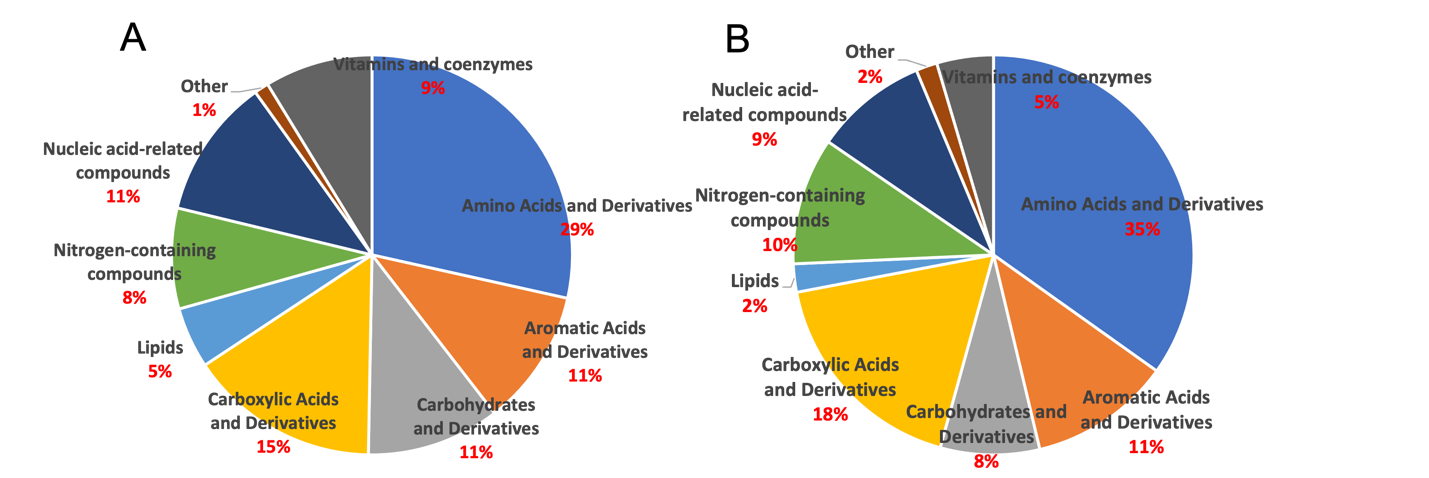
***

### ***Table S1: Detailed information of metabolites reported in this study***

| # | Compounds | Precursor  (m/z) | Product (m/z) | Retention Time (min) | RT Window  (min) | Polarity |
| --- | --- | --- | --- | --- | --- | --- |
| **Amino Acids and Derivatives** | | | | | | |
| 1 | (R)-2-Amino-3-Methoxypropanoic Acid | 120.07 | 73.911, 56.071 | 2.23 | 1 | Positive |
| 2 | 1-Aminocyclopropanecarboxylic Acid | 102.06 | 56.083, 84.03 | 2.18 | 1 | Positive |
| 3 | 1,4-Diaminobutane Dihydrochloride | 89.11 | 72.375, 30.262 | 3.43 | 1 | Positive |
| 4 | 2-Aminoisobutyric Acid | 104.07 | 58.054, 42.083 | 2.12 | 1 | Positive |
| 5 | 2-Methylglutaric Acid | 147.08 | 129.054 | 1.39 | 2.5 | Positive |
| 6 | 2-Oxo-3-Phenylpropanoic Acid | 165.05 | 91.054, 119.071 | 6.5 | 13 | Positive |
| 7 | 3-(4-Hydroxyphenyl)Lactate | 181.04 | 163.054 | 1.53 | 3 | Negative |
| 8 | 3-Hydroxyanthranilate | 154.05 | 136.039 | 6.5 | 13 | Positive |
| 9 | 3-Hydroxyphenylacetate | 153.06 | 125.22 | 1.46 | 2.8 | Positive |
| 10 | 3-Indolepropionic Acid | 190.09 | 130.083, 172.083 | 1.21 | 1 | Positive |
| 11 | 3-Ureidopropionate | 133.06 | 90.054, 115.049 | 1.71 | 1 | Positive |
| 12 | 4-Aminobutanoate | 104.07 | 87.044 | 2.23 | 1 | Positive |
| 13 | 4-Guanidinobutanoate | 146.09 | 87.045, 86.061 | 2.11 | 2 | Positive |
| 14 | 5-Aminolevulinic Acid | 132.06 | 86.06, 114.054 | 2.92 | 3 | Positive |
| 15 | 5-Aminopentanoate | 118.08 | 55.055, 101.06 | 2.13 | 2 | Positive |
| 16 | 5-Hydroxyindoleacetate | 192.07 | 146.111 | 1.33 | 2.5 | Positive |
| 17 | Alanine | 90.15 | 45.222, 44.222 | 2.43 | 3 | Positive |
| 18 | Aminoadipic Acid | 162.08 | 98.125, 144.054 | 2.41 | 1 | Positive |
| 19 | Arginine | 175.15 | 60.056, 70.111 | 4.36 | 3 | Positive |
| 20 | Asparagine | 133.15 | 74.024, 87.111 | 1.93 | 3 | Positive |
| 21 | Aspartic Acid | 134.24 | 74.058, 88.111 | 1.92 | 3 | Positive |
| 22 | Carnosine | 227.11 | 210.087, 156.076 | 3.17 | 1 | Positive |
| 23 | Citrulline | 176.1 | 159.076, 113.071 | 2.96 | 3 | Positive |
| 24 | Creatinine | 114.07 | 44.113 | 1.81 | 2 | Positive |
| 25 | Cys-Gly | 179.05 | 76.022, 162.022 | 6.5 | 13 | Positive |
| 26 | Cysteine | 122.12 | 76.111, 59.111 | 2.28 | 3 | Positive |
| 27 | D-(+)-Galactosamine Hcl | 180.09 | 162.125, 72.071 | 2.87 | 1 | Positive |
| 28 | Dl-Homocysteine | 136.04 | 90.179, 56.036 | 2.04 | 1 | Positive |
| 29 | Dl-Kynurenine | 209.09 | 192.054, 146.083 | 1.89 | 1 | Positive |
| 30 | Ethyl 3-Indoleacetate | 204.1 | 130.14 | 1.34 | 2.5 | Positive |
| 31 | Glutamic Acid | 148.06 | 130.05, 102.183 | 3.04 | 3 | Positive |
| 32 | Glutamine | 147.09 | 130.026, 84.045 | 2.9 | 3 | Positive |
| 33 | Glutathione | 308.09 | 179.05, 162.024 | 2.24 | 2 | Positive |
| 34 | Glycine | 76.12 | 47.222, 30.333 | 2.78 | 3 | Positive |
| 35 | Glycocyamine | 118.06 | 91.125, 100.125 | 2.11 | 1 | Positive |
| 36 | Hippurate | 180.07 | 105.034 | 1.4 | 1 | Positive |
| 37 | Histidine | 156.12 | 110.111, 95.111 | 3.65 | 4 | Positive |
| 38 | Histidinol | 142.1 | 124.087, 81.045 | 2.43 | 1 | Positive |
| 39 | Homocysteine | 136.04 | 56.051, 90.038 | 1.93 | 1 | Positive |
| 40 | Indoxyl Sulfate | 212 | 79.988, 132.054 | 2.16 | 1 | Negative |
| 41 | Indoxyl Sulfate Potassium Salt | 212 | 79.958, 132.045 | 1.21 | 1 | Negative |
| 42 | L-Carnosine | 227.11 | 209.75, 81.095 | 2.41 | 1 | Positive |
| 43 | L-Citrulline | 174.1 | 131.071, 126.929 | 2.41 | 1 | Negative |
| 44 | L-Glutathione Reduced | 308.09 | 179.054, 161.982 | 1.96 | 2 | Positive |
| 45 | L-Histidinol Dihydrochloride | 142.1 | 124.083, 81.065 | 2.44 | 1 | Positive |
| 46 | L-Homoserine | 120.07 | 74.071, 88.042 | 2.27 | 1 | Positive |
| 47 | L(-)-Pipecolinic Acid | 130.09 | 83.75, 56.179 | 2.1 | 1 | Positive |
| 48 | Lauroylcarnitine | 344.28 | 85.029 | 6.5 | 13 | Positive |
| 49 | Leucine/Isoleucine | 132.15 | 44.183, 86.183 | 1.98 | 3 | Positive |
| 50 | Lysine | 147.15 | 84.183, 130.165 | 4.5 | 4 | Positive |
| 51 | Melatonin | 233.13 | 174.091 | 1.19 | 1 | Positive |
| 52 | Methionine | 150.12 | 133.054, 104.111 | 1.99 | 3 | Positive |
| 53 | Methyl 4-Aminobutyrate Hcl | 118.09 | 101.155, 59.054 | 2.11 | 1 | Positive |
| 54 | Methyl Indole-3-Acetate | 190.09 | 172.12, 130.16 | 1.4 | 2.5 | Positive |
| 55 | N-Acetyl-Glycine | 118.05 | 75.982, 43.054 | 1.72 | 2 | Positive |
| 56 | N-Acetyl-L-Cysteine | 164.04 | 122.042, 58.97 | 1.96 | 1 | Positive |
| 57 | N-Acetyl-L-Leucine | 174.11 | 128.21, 156.12 | 1.54 | 3 | Positive |
| 58 | N-Acetyl-Ornithine | 175.11 | 115.086, 158.08 | 2.25 | 2 | Positive |
| 59 | N-Acetyl-Serotonin | 219.11 | 160.18, 202.16 | 1.52 | 3 | Positive |
| 60 | N,N-Dimethylglycine Hydrochloride | 104.07 | 58.125, 44.083 | 2.04 | 1 | Positive |
| 61 | O-Acetyl-L-Carnitine HCl | 204.12 | 85.071, 145.054 | 2.12 | 1 | Positive |
| 62 | O-Methyl-L-Serine Hydrochloride | 120.07 | 78.929, 74.179 | 1.46 | 1 | Positive |
| 63 | Ornithine | 133.1 | 116.071, 70.066 | 4.53 | 4 | Positive |
| 64 | Phenylac-Gln-Oh | 265.12 | 130.125, 91.054 | 1.26 | 1 | Positive |
| 65 | Phenylalanine | 166.15 | 120.111, 103.111 | 1.91 | 3 | Positive |
| 66 | Phenylpyruvate | 163.04 | 91.055 | 6.5 | 13 | Negative |
| 67 | Phosphoserine | 181.07 | 163.208, 67.196 | 1.32 | 1 | Positive |
| 68 | Proline | 116.15 | 70.183 | 2.26 | 3 | Positive |
| 69 | Pyridoxal | 168.07 | 150.12 | 1.78 | 3 | Positive |
| 70 | Sarcosine | 90.06 | 59.013, 58.066 | 2.7 | 2 | Positive |
| 71 | Serine | 106.12 | 60.183, 42.111 | 2.88 | 3 | Positive |
| 72 | Spermidine | 146.17 | 72, 112.226 | 5.02 | 2 | Positive |
| 73 | Spermine | 203.22 | 112.112, 129.138 | 6.5 | 13 | Positive |
| 74 | Taurine | 126.02 | 78.985, 64.97 | 1.97 | 1 | Positive |
| 75 | Threonine | 120.12 | 56.165, 74.183 | 2.8 | 3 | Positive |
| 76 | Trans-3-Indoleacrylic Acid | 188.07 | 114.994, 170.214 | 1.25 | 1 | Positive |
| 77 | Tryptophan | 205.15 | 146.06, 187.968 | 1.86 | 3 | Positive |
| 78 | Tyrosine | 182.15 | 136.183, 165.196 | 2.12 | 3 | Positive |
| 79 | Urea | 61.04 | 43.054, 46 | 1.5 | 1 | Positive |
| 80 | Urocanate | 139.05 | 121.039, 93.045 | 1.58 | 1 | Positive |
| 81 | Valine | 118.21 | 72.183, 55.165 | 2.15 | 3 | Positive |
| **Aromatic Acids and Derivatives** | | | | | | |
| 82 | 2-Hydroxypyridine | 96.04 | 78, 51 | 1.48 | 1 | Positive |
| 83 | 2,5-Dihydroxybenzoic Acid | 155.03 | 137.071, 81.071 | 6.5 | 13 | Positive |
| 84 | 2,6-Dihydroxypyridine HCl | 112.04 | 94.054, 66 | 1.54 | 1 | Positive |
| 85 | 3-Dehydroshikimate | 171.03 | 109.032, 127.042 | 6.5 | 13 | Negative |
| 86 | 3-Hydroxybenzoate | 137.05 | 93.111 | 1.46 | 2.5 | Negative |
| 87 | 3-Methoxy-4-Hydroxymandelate | 197.04 | 137.111 | 1.58 | 3 | Negative |
| 88 | 3-Methyl-2-Oxindole | 148.1 | 130.111 | 1.4 | 2.8 | Positive |
| 89 | 3,4,5-Trimethoxycinnamic Acid | 239.09 | 169.125, 197.054 | 2.4 | 1 | Positive |
| 90 | 4-Aminobenzoate | 138.06 | 77.093, 94.066 | 2.13 | 3 | Positive |
| 91 | 4-Hydroxybenzoate (P-Salicylic Acid) | 137.02 | 93.034 | 1.28 | 1 | Negative |
| 92 | 4-Methylcatechol | 123.07 | 95.04 | 1.33 | 2.5 | Negative |
| 93 | 4-Quinolinecarboxylic Acid | 174.06 | 146.15 | 1.67 | 3 | Positive |
| 94 | Allantoin | 157.03 | 97.003, 41.999 | 1.7 | 1 | Negative |
| 95 | Anthranilate | 138.05 | 92.05, 120.044 | 6.5 | 13 | Positive |
| 96 | Benzoic Acid | 123.04 | 79.054, 77 | 6.5 | 13 | Positive |
| 97 | Catechol | 109.03 | 65.002, 41.007 | 1.21 | 1 | Negative |
| 98 | Dihydroorotate | 157.03 | 113.038, 42.001 | 1.86 | 1 | Negative |
| 99 | Dl-2-Phenylpropionic Acid | 151.08 | 105.196, 76.911 | 6.5 | 13 | Positive |
| 100 | Ferulate | 195.07 | 145.032 | 1.19 | 1 | Positive |
| 101 | Homogentisate | 169.07 | 123.111 | 1.66 | 3 | Positive |
| 102 | Homovanillate | 183.07 | 137.04 | 1.48 | 2.5 | Positive |
| 103 | Hydrocinnamic Acid | 151.08 | 105.196, 133.244 | 1.48 | 1 | Positive |
| 104 | Hydroquinone | 111.04 | 54.982, 69.054 | 1.55 | 1 | Positive |
| 105 | Isonicotinic Acid | 124.04 | 78.071, 96.083 | 1.2 | 1 | Positive |
| 106 | Mandelic Acid | 151.04 | 107.05 | 1.38 | 1 | Negative |
| 107 | Ortho-Hydroxyphenylacetic Acid | 153.05 | 107.054, 77 | 1.36 | 1 | Positive |
| 108 | Phenol | 95.05 | 77.071, 51.071 | 6.5 | 13 | Positive |
| 109 | Phenylacetic Acid | 135.06 | 91.155 | 1.34 | 2.5 | Negative |
| 110 | Quinate | 191.06 | 93.035, 85.03 | 2.03 | 2 | Negative |
| 111 | Quinolinate | 166.02 | 122.023, 78.035 | 3.05 | 3 | Negative |
| 112 | Resorcinol Monoacetate | 153.07 | 111.054 | 1.38 | 2.5 | Positive |
| 113 | Salicylamide | 138.08 | 121.054 | 1.45 | 2.5 | Positive |
| 114 | Salicylate | 139.07 | 121.054 | 1.61 | 3 | Positive |
| 115 | Shikimate | 173.05 | 73.03, 93.035 | 1.95 | 1 | Negative |
| 116 | Trans-Cinnamaldehyde | 133.1 | 115.208, 105.107 | 1.13 | 2.2 | Positive |
| **Carbohydrates and Derivatives** | | | | | | |
| 117 | UDP-Glucose | 565.05 | 323.027 | 3 | 2 | Negative |
| 118 | UDP-N-Acetylglucosamine | 606.08 | 282.039, 384.986 | 6.5 | 13 | Negative |
| 119 | D-(-)-Fructose | 181.07 | 163.125, 135.083 | 1.6 | 1 | Positive |
| 120 | D-(+)-Glucosamine Hydrochloride | 180.09 | 161.982, 72.071 | 2.84 | 1 | Positive |
| 121 | D-(−)-Arabinose | 151.06 | 133.042, 123.083 | 6.5 | 13 | Positive |
| 122 | D-Fructose-6-Phosphate Disodium Salt | 259.02 | 192.988, 96.899 | 1.92 | 1 | Negative |
| 123 | D-Glucuronic Acid Sodium Salt Monohydrate | 195.05 | 167.143, 95 | 1.29 | 1 | Positive |
| 124 | D-Gulonic Acid Gama-Lactone | 177.02 | 89.071 | 1.76 | 3.5 | Negative |
| 125 | D-Mannosamine HCl | 180.09 | 162.101, 72.071 | 2.77 | 1 | Positive |
| 126 | D-Psicose | 181.02 | 163.054, 149.111 | 1.33 | 2.5 | Positive |
| 127 | Erythritol | 123.07 | 43.019, 87.045 | 1.86 | 3 | Positive |
| 128 | Fructose 6-Phosphate | 259.03 | 96.969, 79.143 | 4.38 | 4 | Negative |
| 129 | Gluconate | 195.05 | 129.018, 75.009 | 2.1 | 1 | Negative |
| 130 | Gluconolactone | 179.05 | 115.038, 73.029 | 2.1 | 1 | Positive |
| 131 | Glucosamine-6-Phosphate | 260.05 | 98.055, 84.045 | 6.5 | 13 | Positive |
| 132 | Glucose | 179.06 | 71.014, 59.014 | 2.36 | 3 | Negative |
| 133 | Glucose-6-Phosphate | 259.91 | 199.071, 97 | 5.12 | 4 | Negative |
| 134 | Glyceraldehyde | 91.04 | 73.028, 61.028 | 6.5 | 13 | Positive |
| 135 | Glyceraldehyde-3-Phosphate | 169 | 96.969, 78.959 | 6.5 | 13 | Negative |
| 136 | Glycerate | 105.03 | 59.014, 56.998, 59.014, 56.998 | 2.14 | 3 | Negative |
| 137 | Glycerol Monooleate | 357.3 | 265.321, 265.321 | 1.51 | 2 | Positive |
| 138 | Glycerol Monopalmitate | 331.28 | 313.256, 239.137 | 1.06 | 1 | Positive |
| 139 | Glycerol Monostearate | 357.3 | 341.095, 337.095 | 6.5 | 13 | Negative |
| 140 | L-Gulonic Acid Γ-Lactone | 179.06 | 100.988, 78.792 | 1.48 | 1 | Positive |
| 141 | L-Sorbose | 181.04 | 149.071 | 1.39 | 2.5 | Positive |
| 142 | Maltose | 341.11 | 254.97, 321.071 | 2.99 | 1 | Negative |
| 143 | Mannitol | 183.08 | 165.054 | 2.2 | 4 | Positive |
| 144 | Mannose | 181.08 | 163.125 | 1.36 | 2.5 | Positive |
| 145 | Meso-Erythritol | 123.07 | 77.071, 79.071 | 6.5 | 13 | Positive |
| 146 | Myo-Inositol | 181.07 | 133.111, 163.071, 59.014, 161.044 | 1.34 | 2.5 | Positive |
| 147 | N-Acetyl-D-Glucosamine | 222.09 | 126.05, 138.053 | 1.85 | 1 | Positive |
| 148 | Ribitol | 153.08 | 135.054 | 2.04 | 4 | Positive |
| 149 | Sn-Glycerol-3-Phosphate (Cyclohexyl Ammonium Salt Hydrate) | 173.02 | 79.071, 63.97 | 1.42 | 2 | Positive |
| 150 | Stachyose | 689.21 | 527.153 | 3.54 | 1 | Positive |
| 151 | Sucrose | 341.09 | 179.026 | 2.58 | 4 | Negative |
| 152 | Udp-Α-D-Glucose (Sodium Salt) | 565.05 | 323.22, 96.929 | 2.92 | 2 | Negative |
| **Carboxylic Acids and Derivatives** | | | | | | |
| 153 | 2-Aminoethyl Phosphonate | 126.03 | 109.005 | 6.5 | 13 | Positive |
| 154 | 2-Furoic Acid | 113.02 | 95, 67 | 6.5 | 13 | Positive |
| 155 | 2-Hydroxyisobutyric Acid | 105.06 | 77.018, 59.155 | 1.2 | 1 | Positive |
| 156 | 2-Ketobutyric Acid Sodium Salt | 125.02 | 80.929, 97.131 | 2.45 | 2 | Positive |
| 157 | 2-Methylmaleate/Itaconate | 129.04 | 41.222, 85.111 | 1.47 | 2.5 | Negative |
| 158 | 2-Oxoadipate | 161.09 | 129.111 | 1.59 | 3 | Positive |
| 159 | 2-Oxobutanoate | 101.02 | 73.032, 55.019 | 1.87 | 2 | Negative |
| 160 | 3-Phosphoglyceric Acid | 184.99 | 78.959, 96.969 | 3.05 | 3 | Negative |
| 161 | 5-Aminolevulinic Acid Hydrochloride | 132.07 | 114.054, 86.054 | 2.34 | 1 | Positive |
| 162 | 6-Carboxyhexanoate | 161.08 | 125.06, 69.071 | 6.5 | 13 | Positive |
| 163 | Acetoacetate | 103.18 | 71.183 | 1.87 | 3 | Positive |
| 164 | Acetyl Phosphate | 141.1 | 109.111 | 1.37 | 2.5 | Positive |
| 165 | Aconitate | 175.02 | 111.007 | 4.94 | 3 | Positive |
| 166 | Adipic Acid | 147.07 | 128.911, 110.923 | 6.5 | 13 | Positive |
| 167 | Alpha-Ketoglutaric Acid | 145.01 | 57.035, 101.024 | 1.91 | 3 | Negative |
| 168 | Azelaic Acid | 189.1 | 171.111 | 1.36 | 2.5 | Positive |
| 169 | Citrate | 193.03 | 139.002, 129.017 | 2.3 | 4 | Positive |
| 170 | Dl-Isocitric Acid Trisodium Salt Hydrate | 191.02 | 94.845, 79.905 | 6.5 | 13 | Negative |
| 171 | DL-Mevalonolactone | 131.07 | 113.196, 69.083 | 1.29 | 1 | Positive |
| 172 | Ethanolamine Phosphate | 142.03 | 44.051 | 2.92 | 3 | Positive |
| 173 | Fumarate | 115 | 71.014, 27.024 | 1.73 | 3 | Negative |
| 174 | Galactarate | 209.03 | 71.014, 59.014 | 2.32 | 1 | Negative |
| 175 | Glutaric Acid | 133.05 | 115.161, 87.161 | 6.5 | 13 | Positive |
| 176 | Guanidinoacetate | 118.06 | 72.056, 76.396 | 2.13 | 2 | Positive |
| 177 | Hexanoic Acid | 117.09 | 89.143, 43.155 | 1.52 | 1 | Positive |
| 178 | Isobutyric Acid | 89.06 | 43.083, 71.071 | 6.5 | 13 | Positive |
| 179 | Isocitric Acid | 191.03 | 87.009, 111.009 | 2.02 | 1 | Negative |
| 180 | Isopentyl Pyrophosphate | 245 | 78.959 | 6.5 | 13 | Negative |
| 181 | Isovaleric Acid | 103.08 | 43.083, 61.071 | 1.41 | 1 | Positive |
| 182 | L-Threonic Acid Calcium Salt | 135.03 | 74.97, 89.214 | 1.75 | 1 | Negative |
| 183 | Lactate | 89.03 | 71.015, 43.02 | 1.66 | 2 | Negative |
| 184 | Malate | 133.02 | 71.014, 115.003 | 2.16 | 3 | Negative |
| 185 | Maleic Acid | 117.02 | 99, 71 | 6.5 | 13 | Positive |
| 186 | Malonic Acid Disodium Salt | 103 | 58.988, 74.97 | 1.6 | 1 | Negative |
| 187 | Methylmalonate | 117.02 | 73.03 | 6.5 | 13 | Negative |
| 188 | Mevalolactone | 131.07 | 71.05, 69.071 | 6.5 | 13 | Positive |
| 189 | Mono-Methyl Glutarate | 147.09 | 129.111 | 1.36 | 2.5 | Positive |
| 190 | Monomethyl Glutarate | 145.05 | 95, 113.054 | 1.28 | 1 | Negative |
| 191 | Nicotinate | 124.04 | 80.05, 78.034 | 1.62 | 1 | Positive |
| 192 | Oxalic Acid | 91 | 65.071, 72.917 | 0.5 | 1 | Positive |
| 193 | Oxaloacetic Acid | 131.01 | 87.008, 59.015 | 1.42 | 2 | Negative |
| 194 | Pentanoate (Valeric Acid) | 103.08 | 75.045, 57.035 | 3.02 | 3 | Positive |
| 195 | Phospho(Enol)Pyruvic Acid | 169 | 122.98, 150.978 | 6.6 | 13 | Positive |
| 196 | Phospho(Enol)Pyruvic Acid Monopotassium Salt | 169 | 151.054, 133.054 | 2.06 | 1 | Positive |
| 197 | Phosphocholine | 184.07 | 60.081, 125 | 3.13 | 2 | Positive |
| 198 | Propionate | 73.03 | 41.004, 55.019 | 6.5 | 13 | Negative |
| 199 | Pyruvate | 87.02 | 43.018, 86.556 | 1.68 | 3 | Negative |
| 200 | Sorbate | 113.06 | 67.056, 95.05 | 1.25 | 2 | Positive |
| 201 | Suberic Acid | 175.1 | 157.111 | 1.34 | 2.5 | Positive |
| 202 | Succinic Acid | 117.03 | 73.03, 99.009 | 1.61 | 3 | Negative |
| 203 | Trans-Aconitic Acid | 175.02 | 139, 69.042 | 6.5 | 13 | Positive |
| **Lipids** | | | | | | |
| 204 | 2-Hydroxyoctanoic Acid | 161.11 | 81, 65.97 | 1.53 | 1 | Positive |
| 205 | Cholestenone | 385.35 | 109.054, 367.333 | 1.25 | 1 | Positive |
| 206 | Cortisol | 363.22 | 83.052, 121.063 | 1.21 | 1 | Positive |
| 207 | Deoxycholic Acid | 227.2 | 113, 95.929 | 6.5 | 13 | Negative |
| 208 | Elaidic Acid | 281.25 | 131, 229.071 | 6.5 | 13 | Negative |
| 209 | Ethanolamine HCl | 62.06 | 44.071, 47.054 | 2.21 | 1 | Positive |
| 210 | Ethanolamine | 62.06 | 44.051 | 2.41 | 2 | Positive |
| 211 | Ethylene Glycol | 63.04 | 46, 45.071 | 1.45 | 2 | Positive |
| 212 | Glycerol | 93.06 | 75.042, 57.042 | 1.5 | 2 | Positive |
| 213 | Glycocholate | 466.31 | 430.294 | 1.33 | 1 | Positive |
| 214 | Hexadecanoic Acid | 257.25 | 178.958, 100.929 | 1.58 | 1 | Positive |
| 215 | Linoleic Acid (Sodium Salt) | 279.23 | 193.042, 258.958 | 6.5 | 13 | Negative |
| 216 | Myristic Acid | 227.2 | 160.982, 95 | 6.5 | 13 | Negative |
| 217 | Oleic Acid Sodium Salt | 281.25 | 131.071, 19.25 | 6.5 | 13 | Negative |
| 218 | Stearic Acid Methyl Ester | 297.28 | 230.899, 277.042 | 6.5 | 13 | Negative |
| 219 | Α-Linolenic Acid (Sodium Salt) | 277.22 | 275.25, 259.208 | 1.07 | 1 | Negative |
| **Nitrogen-containing compounds** | | | | | | |
| 220 | (S)-Nicotine | 163.13 | 84.08, 106.064 | 2.43 | 3 | Positive |
| 221 | 2-Aminophenol | 110.06 | 92.05, 65.04 | 6.5 | 13 | Positive |
| 222 | 4-Hydroxy-3-Methoxyphenylglycol | 455.2 | 437.155 | 1.46 | 2.8 | Positive |
| 223 | Acetylcholine | 146.12 | 43.018, 87.045 | 1.62 | 2 | Positive |
| 224 | Cadaverine | 103.12 | 86.097 | 3.23 | 1 | Positive |
| 225 | Caffeine | 195.09 | 138.066, 110.072 | 1.27 | 1 | Positive |
| 226 | Choline Chloride | 104.11 | 57.78, 44.119 | 2.14 | 1 | Positive |
| 227 | Dopamine | 154.09 | 137.06 | 2.08 | 2 | Positive |
| 228 | Histamine | 112.09 | 68.052, 95.062 | 2.39 | 1 | Positive |
| 229 | Indole-3-Acetaldehyde | 160.08 | 142.07, 118.11 | 1.93 | 3.8 | Positive |
| 230 | Indole-3-Acetate | 174.05 | 130.064 | 1.23 | 1 | Negative |
| 231 | L-Nicotine | 163.12 | 130.054, 132.06 | 2.13 | 1 | Positive |
| 232 | Noradrenaline | 170.08 | 152.073, 135.046 | 2.13 | 1 | Positive |
| 233 | Octopamine | 154.09 | 136.076, 91.054 | 2.1 | 2 | Positive |
| 234 | Paraxanthine | 179.06 | 164.033, 122.036 | 1.37 | 1 | Negative |
| 235 | Phenethylamine | 122.1 | 105.17 | 1.65 | 3 | Positive |
| 236 | Piperine | 286.14 | 201.054, 115.071 | 1.14 | 1 | Positive |
| 237 | Putrescine | 89.11 | 72.082 | 3.37 | 2 | Positive |
| 238 | Serotonin | 177.1 | 160.075 | 2.03 | 2 | Positive |
| 239 | Serotonin HCl | 177.1 | 160.125, 115.054 | 2.13 | 1 | Positive |
| 240 | Theobromine | 181.07 | 163.125, 135.125 | 1.33 | 1 | Positive |
| 241 | Theophylline | 181.07 | 124.05, 96.056 | 1.34 | 1 | Positive |
| 242 | Trigonelline | 138.06 | 94.065 | 1.94 | 1 | Positive |
| 243 | Tryptamine | 161.11 | 81, 65.97 | 1.46 | 1 | Positive |
| 244 | Tyramine | 138.09 | 77.038, 121.065 | 1.67 | 1 | Positive |
| **Nucleic acid-related compounds** | | | | | | |
| 245 | (CMP) Cytidine Monophosphate | 322.04 | 78.959, 96.968 | 3.75 | 4 | Negative |
| 246 | (Cyclic AMP) Adenosine 3'5'-Cyclic Monophosphate | 330.09 | 136.062, 312.049 | 1.98 | 3 | Positive |
| 247 | (Cyclic CMP) Cytidine 2'3'-Cyclic Monophosphate | 306.05 | 112.05, 178.061 | 2.23 | 3 | Positive |
| 248 | (DAMP) Deoxyadenosine Monophosphate | 330.06 | 134.046, 195.005 | 2.92 | 3 | Negative |
| 249 | (DCMP) 2'-Deoxycytidine 5'-Monophosphate | 308.06 | 95.023, 112.051 | 3.46 | 4 | Positive |
| 250 | (DGDP)2-Deoxyguanosine 5'-Diphosphate | 426.02 | 158.925, 408.01 | 6.5 | 13 | Negative |
| 251 | (DGMP) 2-Deoxyguanosine 5'-Monophosphate | 348.07 | 152.056 | 2.14 | 2 | Positive |
| 252 | (GMP) Guanosine 5'-Monophosphate | 362.05 | 78.959, 210.999 | 2.61 | 2 | Negative |
| 253 | (GTP) Guanosine 5'-Triphosphate | 521.99 | 158.923, 424.004 | 4.21 | 3 | Negative |
| 254 | 2'-Deoxyinosine | 251.08 | 135, 108.071 | 1.58 | 1 | Negative |
| 255 | 3-Methyladenine | 150.08 | 133.12 | 2.24 | 4 | Positive |
| 256 | 3-Methylbutanal | 85.11 | 41.222, 41.165 | 1.47 | 2.5 | Negative |
| 257 | 5-(Hydroxymethyl)Uracil | 143.05 | 125.054, 82.071 | 1.76 | 2 | Positive |
| 258 | 5-Hydroxymethyluracil | 143.04 | 125.034, 82.029 | 2.04 | 4 | Positive |
| 259 | Adenine | 136.05 | 119.035, 94.04 | 1.76 | 3 | Positive |
| 260 | Adenosine | 268.1 | 136.061 | 1.61 | 1 | Positive |
| 261 | Amp | 348.07 | 119.032, 69.035, 136.062 | 3.02 | 4 | Positive |
| 262 | Cytidine | 244.09 | 112.05 | 1.82 | 1 | Positive |
| 263 | Cytosine | 112.04 | 69.045, 95.024 | 1.96 | 3 | Positive |
| 264 | Deoxyadenosine | 252.11 | 136.061 | 1.5 | 1 | Positive |
| 265 | Deoxyguanosine | 268.1 | 135.029, 110.034 | 2.98 | 2 | Positive |
| 266 | Deoxyribose-5-P | 215.02 | 121.026 | 6.5 | 13 | Positive |
| 267 | Glycolaldehyde Dimer | 121.05 | 43.083, 93.054 | 6.5 | 13 | Positive |
| 268 | Guanine | 152.06 | 135.054, 110.071 | 1.96 | 1 | Positive |
| 269 | Guanosine | 284.98 | 152.056 | 1.85 | 1 | Positive |
| 270 | Hypoxanthine | 137.05 | 110.035, 119.035 | 1.62 | 1 | Positive |
| 271 | Isovaleraldehyde | 87.08 | 45.125, 43.054 | 2.28 | 1 | Positive |
| 272 | Pterin | 164.06 | 119.034, 147.03 | 6.5 | 13 | Positive |
| 273 | Purine | 121.15 | 94.04 | 1.84 | 2 | Positive |
| 274 | Pyruvic Aldehyde | 73.18 | 55.165 | 1.33 | 2.5 | Positive |
| 275 | Pyruvic Aldehyde Sln Aq 35-45% | 73.03 | 55.071, 45.083 | 1.53 | 2 | Positive |
| 276 | Thymine | 127.04 | 54.034, 110.143 | 6.5 | 13 | Positive |
| 277 | Uracil | 111.03 | 41.998, 111.019 | 1.98 | 3 | Negative |
| 278 | Urate | 167.02 | 41.999, 124.015 | 1.67 | 1 | Negative |
| 279 | Uridine | 245.07 | 113.035 | 1.63 | 2 | Positive |
| 280 | Xanthine | 153.04 | 110.035, 55.03 | 6.5 | 13 | Positive |
| 281 | Xanthosine Dihydrate | 285.08 | 153.095, 135.899 | 1.73 | 1 | Positive |
| **Other** | | | | | | |
| 282 | 3-Methyl-2-Oxovaleric Acid | 131.1 | 78.111 | 1.3 | 2.5 | Positive |
| 283 | 4-Methyl-2-Oxovaleric Acid | 129.08 | 57.222 | 2.16 | 4 | Negative |
| 284 | Dimethyl Sulfone | 95.02 | 64.917, 62.97 | 1.49 | 2 | Positive |
| 285 | Thiourea | 77.02 | 59.991 | 1.44 | 2 | Positive |
| **Vitamins and coenzymes** | | | | | | |
| 286 | (ADP) Adenosine 3'5'-Diphosphate | 426.02 | 134.046, 328.044 | 3.5 | 4 | Negative |
| 287 | (ATP) Adenosine 5'-Triphosphate | 505.98 | 408.264, 428.04 | 4.99 | 4 | Negative |
| 288 | (FAD) Flavin Adenine Dinucleotide | 784.15 | 180.992, 437.085, 346.056 | 3.14 | 3 | Negative |
| 289 | (GDP) Guanosine 5'-Diphosphate | 442.01 | 150.042, 158.925 | 5 | 4 | Negative |
| 290 | (NAD) Nicotinamide Adenine Dinucleotide | 664.12 | 428.04, 524.057 | 3.76 | 4 | Positive |
| 291 | (NADH) Nicotinamide Adenine Dinucleotide (Reduced) | 664.11 | 524.055, 542.065 | 2.75 | 2 | Positive |
| 292 | (NADPH) Nicotinamide Adenine Dinucleotide Phosphate (Reduced) | 746.1 | 136.061, 150.055 | 3.35 | 3 | Positive |
| 293 | (PRPP) Phosphoribosyl Pyrophosphate | 388.94 | 176.935, 290.966 | 6.5 | 13 | Negative |
| 294 | 1-Methylnicotinamide Chloride | 173.05 | 155.125, 112.083 | 6.5 | 13 | Positive |
| 295 | Acetoacetyl-Coa | 850.13 | 766.105, 408.012 | 6.5 | 13 | Negative |
| 296 | Acetyl-Coa | 810.13 | 428.035, 303.137 | 6.5 | 13 | Positive |
| 297 | Adenosine Phosphosulfate | 428.02 | 158.044, 234.996 | 6.5 | 13 | Positive |
| 298 | Biotin | 245.1 | 227.1 | 1.5 | 3 | Positive |
| 299 | Coenzyme A | 768.12 | 426.014 | 6.5 | 13 | Positive |
| 300 | Dethiobiotin | 215.14 | 197.11, 179.17 | 1.56 | 3 | Positive |
| 301 | Flavine Mononucleotide | 455.1 | 78.959, 96.969 | 2 | 1 | Negative |
| 302 | Folic Acid | 442.15 | 295.094 | 2 | 1 | Positive |
| 303 | Glutathione Disulfide | 613.16 | 484.115, 355.073 | 3.79 | 2 | Positive |
| 304 | Lipoamide | 206.07 | 161.044, 189.037 | 1.2 | 1 | Positive |
| 305 | Malonyl-Coa | 854.12 | 428.036, 347.127 | 6.5 | 13 | Positive |
| 306 | Nicotinamide | 123.06 | 80.045 | 1.41 | 1 | Positive |
| 307 | Phosphocreatine | 210.03 | 78.959 | 2.89 | 3 | Negative |
| 308 | Propionyl-Coa | 822.13 | 408.01, 475.07 | 6.5 | 13 | Negative |
| 309 | Pyridoxine | 170.08 | 152.07 | 1.61 | 1 | Positive |
| 310 | Riboflavin | 377.14 | 243.089, 172.087 | 2.42 | 1 | Positive |
| 311 | S-Adenosyl-Methionine | 399.15 | 136.06, 250.092 | 3.4 | 1 | Positive |
| 312 | Sodium D-Pantothenate | 218.1 | 87.946, 146.244 | 1.38 | 1 | Negative |
| 313 | Succinyl-Coa | 866.12 | 408.009, 426.022 | 6.5 | 13 | Negative |
| 314 | Tetrahydrofolate | 446.18 | 299.124, 166.072 | 6.5 | 13 | Positive |
| 315 | Thiamine | 265.11 | 144.048, 122.071 | 2.13 | 1 | Positive |

### **Table S2. Internal standards list and the concentration.**

|  | |
| --- | --- |
| **STD** | **Stock Mix Conc. (µM)** |
| 13C, 15N-Aspartic Acid | 681.3 |
| 13C, 15N-Glutamic Acid | 646.5 |
| 13C, 15N-Asparagine | 672.5 |
| 13C, 15N-Serine | 392.0 |
| 13C, 15N-Glutamine | 606.6 |
| 13C, 15N-Histidine | 67.02 |
| 13C, 15N-Glycine | 876.2 |
| 13C, 15N-Threonine | 324.9 |
| 13C, 15N-Alanine | 931.9 |
| 13C, 15N-Arginine | 563.9 |
| 13C, 15N-Tyrosine | 70.30 |
| 13C, 15N-Cystine | 54.14 |
| 13C, 15N-Valine | 287.9 |
| 13C, 15N-Methionine | 141.7 |
| 13C, 15N-Tryptophan | 163.2 |
| 13C, 15N-Phenylalanine | 111.6 |
| 13C, 15N-Isoleucine | 159.3 |
| 13C, 15N-Leucine | 822.9 |

**Table S3.** Distribution of metabolites by classes in target and detected list.

| Compound Class | Target list | Detected |
| --- | --- | --- |
| Amino Acids and Derivatives | 98 | 61 |
| Aromatic Acids and Derivatives | 38 | 20 |
| Carbohydrates and Derivatives | 37 | 14 |
| Carboxylic Acids and Derivatives | 53 | 31 |
| Lipids | 17 | 4 |
| Nitrogen-containing compounds | 28 | 18 |
| Nucleic acid-related compounds | 39 | 16 |
| Other | 4 | 3 |
| Vitamins and coenzymes | 30 | 8 |

Table S4. Significantly changed metabolites among 25 years, 35 years and 45 years old.

| **Number** | **Class** | **Metabolites** | **Average intensity at 25 (years)** | **Average intensity at 35 (years)** | **Average intensity at 45 (years)** |
| --- | --- | --- | --- | --- | --- |
| ***up-up*** | | | | | |
| 1 | Amino Acids and Derivatives | Creatinine | 8.76E+06 | 8.85E+06 | 1.04E+07 |
| ***down-down*** | | | | | |
| 1 | Amino Acids and Derivatives | Dl-Homocysteine | 9.56E+04 | 9.17E+04 | 8.09E+04 |
| 2 | Amino Acids and Derivatives | GLycocyamine | 1.63E+06 | 1.60E+06 | 1.32E+06 |
| 3 | Amino Acids and Derivatives | Histidine | 1.89E+07 | 1.83E+07 | 1.62E+07 |
| 4 | Amino Acids and Derivatives | N-Acetyl-L-Cysteine | 1.45E+06 | 1.45E+06 | 1.39E+06 |
| 5 | Amino Acids and Derivatives | N,N-Dimethylglycine | 1.26E+07 | 1.20E+07 | 1.04E+07 |
| 6 | Amino Acids and Derivatives | Phosphoserine | 8.54E+04 | 7.29E+04 | 5.43E+04 |
| 7 | Amino Acids and Derivatives | Tryptophan | 7.34E+06 | 7.30E+06 | 6.53E+06 |
| 8 | Amino Acids and Derivatives | 2-Aminoisobutyric Acid | 8.44E+06 | 8.07E+06 | 7.14E+06 |
| 9 | Amino Acids and Derivatives | 2-Oxo-3-Phenylpropanoic Acid | 1.14E+06 | 1.10E+06 | 9.77E+05 |
| 10 | Aromatic Acids and Derivatives | Benzoic acid | 6.00E+05 | 5.84E+05 | 5.15E+05 |
| 11 | Aromatic Acids and Derivatives | Dl-2-Phenylpropionic Acid | 6.07E+04 | 5.91E+04 | 5.09E+04 |
| 12 | Aromatic Acids and Derivatives | Hydroquinone | 1.47E+05 | 1.46E+05 | 1.35E+05 |
| 13 | Aromatic Acids and Derivatives | Phenol | 2.92E+05 | 2.88E+05 | 2.57E+05 |
| 14 | Aromatic Acids and Derivatives | Trans-Cinnamaldehyde | 4.14E+05 | 3.94E+05 | 3.30E+05 |
| 15 | Aromatic Acids and Derivatives | 3,4,5-Trimethoxycinnamic Acid | 1.83E+05 | 1.75E+05 | 1.21E+05 |
| 16 | Carbohydrates and Derivatives | Glycerate | 2.41E+05 | 2.29E+05 | 1.61E+05 |
| 17 | Carbohydrates and Derivatives | Meso-Erythritol | 5.98E+05 | 5.67E+05 | 5.04E+05 |
| 18 | Carboxylic Acids and Derivatives | Hexanoic acid | 3.28E+05 | 3.00E+05 | 2.08E+05 |
| 19 | Carboxylic Acids and Derivatives | Lactate | 1.50E+07 | 1.36E+07 | 1.14E+07 |
| 20 | Lipids | Glycerol | 2.12E+04 | 2.11E+04 | 1.95E+04 |
| 21 | Nitrogen-containing compounds | Choline_chloride | 2.42E+07 | 2.30E+07 | 1.99E+07 |
| 22 | Nitrogen-containing compounds | Theobromine | 1.30E+05 | 1.19E+05 | 9.19E+04 |
| 23 | Nitrogen-containing compounds | 2-Aminophenol | 3.28E+05 | 3.25E+05 | 2.95E+05 |
| ***down-up*** | | | | | |
| 1 | Aromatic Acids and Derivatives | 3-Dehydroshikimate | 4.10E+05 | 3.31E+05 | 3.32E+05 |
| 2 | Carboxylic Acids and Derivatives | 2-Oxobutanoate | 1.94E+04 | 1.80E+04 | 2.26E+04 |
| 3 | Vitamins and coenzymes | PRPP | 2.19E+04 | 1.99E+04 | 2.49E+04 |
| ***up-down*** | | | | | |
| 1 | Amino Acids and Derivatives | Methyl 4-Aminobutyrate Hcl | 3.78E+07 | 3.89E+07 | 3.38E+07 |
| 2 | Amino Acids and Derivatives | N-Acetyl-Glycine | 1.03E+06 | 1.07E+06 | 9.43E+05 |
| 3 | Amino Acids and Derivatives | 1,4-Diaminobutane | 2.59E+04 | 2.62E+04 | 2.39E+04 |
| 4 | Amino Acids and Derivatives | 3-Hydroxyanthranilate | 3.16E+06 | 3.22E+06 | 3.00E+06 |
| 5 | Aromatic Acids and Derivatives | Allantoin | 6.54E+04 | 7.42E+04 | 5.85E+04 |
| 6 | Aromatic Acids and Derivatives | Anthranilate | 6.99E+05 | 8.93E+05 | 8.57E+05 |
| 7 | Aromatic Acids and Derivatives | Salicylate | 1.02E+06 | 1.02E+06 | 9.33E+05 |
| 8 | Aromatic Acids and Derivatives | 4-Aminobenzoate | 1.25E+05 | 2.84E+05 | 2.74E+05 |
| 9 | Carbohydrates and Derivatives | D-Glucuronic Acid | 8.65E+04 | 9.35E+04 | 5.16E+04 |
| 10 | Carboxylic Acids and Derivatives | Adipic acid | 8.46E+05 | 8.76E+05 | 7.56E+05 |
| 11 | Carboxylic Acids and Derivatives | Glutaric acid | 1.54E+06 | 1.68E+06 | 1.44E+06 |
| 12 | Carboxylic Acids and Derivatives | Trans-Aconitic Acid | 2.05E+05 | 2.06E+05 | 1.87E+05 |
| 13 | Aromatic Acids and Derivatives | 2,5-Dihydroxybenzoic Acid | 3.67E+07 | 3.70E+07 | 3.42E+07 |
| 14 | Lipids | Ethylene Glycol | 4.67E+04 | 4.68E+04 | 4.21E+04 |
| 15 | Nitrogen-containing compounds | Trigonelline | 4.55E+05 | 1.36E+06 | 1.29E+06 |
| 16 | Nitrogen-containing compounds | Tyramine | 3.97E+04 | 7.00E+04 | 6.54E+04 |
| 17 | Nucleic acid-related compounds | Guanine | 1.71E+06 | 1.87E+06 | 1.37E+06 |
| 18 | Vitamins and coenzymes | 1-Methylnicotinamide Chloride | 2.01E+05 | 2.01E+05 | 1.83E+05 |

Table S5. Integrated statistical analysis table for metabolites across serum/plasma comparison, gender impact, and age effect

| Compound | Specimen type | | | | Sex | | | | Age | | | |
| --- | --- | --- | --- | --- | --- | --- | --- | --- | --- | --- | --- | --- |
|  | VIP | Regression significance (p-value after FDR correction) | Foldchange  (Plasma/Serum) | Student t-test (p-value) | VIP | Regression significance (p-value after FDR correction) | Foldchange  (Female/Male) | Student t-test (p-value) | VIP | Regression significance (p-value after FDR correction) | Student t-test (p-value, 25 vs 35) | Student t-test (p-value, 35 vs 45) |
| (S)-Nicotine | 0.79 | 1.00E+00 | 0.88 | 1.98E-02 | 0.54 | 1.00E+00 | 0.92 | 1.66E-01 | 1.01 | 1.00E+00 | 8.29E-01 | 7.86E-01 |
| 1-Aminocyclopropanecarboxylic_acid | 0.75 | 1.00E+00 | 1.07 | 2.61E-02 | 2 | 3.26E-06 | 0.86 | 1.37E-07 | 0.86 | 1.00E+00 | 8.56E-01 | 2.18E-01 |
| 1-Methylnicotinamide_chloride | 0.91 | 9.16E-01 | 0.93 | 6.97E-03 | 1.67 | 3.69E-04 | 1.13 | 1.21E-05 | 1.13 | 1.00E+00 | 9.87E-01 | 7.85E-03 |
| 1,4-Diaminobutane | 0.48 | 1.00E+00 | 0.96 | 1.59E-01 | 1.06 | 2.51E-01 | 1.08 | 6.07E-03 | 1.08 | 1.00E+00 | 9.55E-01 | 9.00E-03 |
| 2-Aminoisobutyric_acid | 0.8 | 1.00E+00 | 0.9 | 1.83E-02 | 1.06 | 1.48E-01 | 1.15 | 6.40E-03 | 1.15 | 2.91E-01 | 8.50E-01 | 3.95E-02 |
| 2-Aminophenol | 0.01 | 1.00E+00 | 0.99 | 9.67E-01 | 0.96 | 1.00E+00 | 1.07 | 1.36E-02 | 1.07 | 5.03E-01 | 9.55E-01 | 5.31E-03 |
| 2-ketobutyric_acid | 0.03 | 1.00E+00 | 0.99 | 9.20E-01 | 0.95 | 1.77E-01 | 0.91 | 1.43E-02 | 0.91 | 1.00E+00 | 8.49E-01 | 9.08E-01 |
| 2-Methylglutaric_aicd | 0.09 | 1.00E+00 | 0.99 | 8.02E-01 | 0.58 | 1.00E+00 | 1.05 | 1.38E-01 | 1.05 | 1.00E+00 | 9.71E-01 | 3.38E-01 |
| 2-Methylmaleate/Itaconate | 0.62 | 1.00E+00 | 0.92 | 6.72E-02 | 0.02 | 1.00E+00 | 1 | 9.63E-01 | 1 | 1.00E+00 | 8.29E-01 | 7.67E-01 |
| 2-Oxo-3-phenylpropanoic_acid | 0.8 | 1.00E+00 | 0.91 | 1.74E-02 | 0.15 | 1.00E+00 | 1.04 | 7.11E-01 | 1.04 | 1.00E+00 | 8.56E-01 | 3.95E-02 |
| 2-Oxobutanoate | 0.56 | 1.00E+00 | 0.9 | 9.68E-02 | 0.18 | 1.00E+00 | 0.98 | 6.42E-01 | 0.98 | 1.00E+00 | 8.49E-01 | 3.95E-02 |
| 2,5-Dihydroxybenzoic_acid | 0.94 | 7.70E-01 | 0.95 | 5.14E-03 | 1.08 | 3.21E-01 | 1.06 | 5.17E-03 | 1.06 | 5.92E-01 | 9.67E-01 | 2.24E-03 |
| 3-(4-hydroxyphenyl)lactate | 0.61 | 1.00E+00 | 0.95 | 7.31E-02 | 0.07 | 1.00E+00 | 1 | 8.64E-01 | 1 | 1.00E+00 | 8.18E-01 | 4.55E-01 |
| 3-Dehydroshikimate | 0.19 | 1.00E+00 | 0.94 | 5.78E-01 | 0.13 | 1.00E+00 | 0.98 | 7.43E-01 | 0.98 | 4.96E-01 | 4.59E-02 | 9.76E-01 |
| 3-Hydroxyanthranilate | 0.5 | 1.00E+00 | 0.96 | 1.43E-01 | 1.39 | 9.07E-02 | 1.08 | 3.10E-04 | 1.08 | 1.00E+00 | 8.56E-01 | 2.89E-02 |
| 3-Indolepropionic_aid | 1.15 | 7.48E-02 | 0.94 | 5.90E-04 | 1.67 | 4.66E-03 | 0.93 | 1.31E-05 | 0.93 | 1.00E+00 | 9.87E-01 | 9.04E-01 |
| 3-Methoxy-4-hydroxymandelate | 0.47 | 1.00E+00 | 1.02 | 1.65E-01 | 0.19 | 1.00E+00 | 0.99 | 6.34E-01 | 0.99 | 1.00E+00 | 8.29E-01 | 6.51E-01 |
| 3-Methyladenine | 0.44 | 1.00E+00 | 1.04 | 2.01E-01 | 0.15 | 1.00E+00 | 1 | 6.94E-01 | 1 | 4.89E-01 | 8.49E-01 | 2.14E-01 |
| 3-Ureidopropionate | 0.41 | 1.00E+00 | 0.94 | 2.25E-01 | 0.49 | 1.00E+00 | 1.06 | 2.09E-01 | 1.06 | 1.00E+00 | 9.55E-01 | 1.13E-01 |
| 3,4,5-Trimethoxycinnamic_acid | 1.06 | 1.00E+00 | 0.76 | 1.70E-03 | 2.03 | 2.15E-07 | 1.83 | 7.45E-08 | 1.83 | 1.00E+00 | 9.55E-01 | 1.13E-02 |
| 4-Aminobenzoate | 0.13 | 1.00E+00 | 0.98 | 7.13E-01 | 0.02 | 1.00E+00 | 0.97 | 9.65E-01 | 0.97 | 2.67E-01 | 2.56E-05 | 9.03E-01 |
| 4-Aminobutanoate | 0.61 | 1.00E+00 | 0.93 | 7.24E-02 | 1.09 | 2.92E-01 | 1.14 | 5.14E-03 | 1.14 | 8.10E-01 | 8.49E-01 | 1.56E-01 |
| 4-Guanidinobutanoate | 0.21 | 1.00E+00 | 0.98 | 5.30E-01 | 3.12 | 1.26E-15 | 0.76 | 3.07E-18 | 0.76 | 1.00E+00 | 2.46E-01 | 2.18E-01 |
| 4-Hydroxy-3-Methoxyphenylglycol | 0.16 | 1.00E+00 | 1.03 | 6.35E-01 | 0.81 | 1.00E+00 | 1.14 | 3.73E-02 | 1.14 | 1.00E+00 | 8.43E-01 | 5.35E-02 |
| 4-Hydroxybenzoate | 0.05 | 1.00E+00 | 1.02 | 8.89E-01 | 1.44 | 1.00E+00 | 4.77 | 1.93E-04 | 4.77 | 1.00E+00 | 7.09E-01 | 3.92E-01 |
| 4-Methyl-2-oxovaleric_aicd | 0.31 | 1.00E+00 | 0.98 | 3.58E-01 | 0.4 | 1.00E+00 | 1.05 | 3.02E-01 | 1.05 | 1.00E+00 | 1.52E-01 | 7.75E-01 |
| 5-Aminolevulinic_acid | 0.87 | 1.00E+00 | 0.92 | 9.76E-03 | 0.43 | 1.00E+00 | 0.97 | 2.69E-01 | 0.97 | 1.00E+00 | 8.89E-01 | 4.32E-01 |
| 5-Aminopentanoate | 0.89 | 1.00E+00 | 0.93 | 8.34E-03 | 0.02 | 1.00E+00 | 0.99 | 9.66E-01 | 0.99 | 1.00E+00 | 8.49E-01 | 3.88E-01 |
| 5-Hydroxyindoleacetate | 0.83 | 9.52E-01 | 0.87 | 1.40E-02 | 0.69 | 1.00E+00 | 0.92 | 7.55E-02 | 0.92 | 1.00E+00 | 9.67E-01 | 7.22E-01 |
| 5-Hydroxymethyluracil | 0.44 | 1.00E+00 | 0.98 | 1.96E-01 | 0.22 | 1.00E+00 | 0.98 | 5.70E-01 | 0.98 | 1.00E+00 | 8.49E-01 | 2.06E-01 |
| Acetylcholine | 0.14 | 1.00E+00 | 1.01 | 6.91E-01 | 3.32 | 5.21E-18 | 0.72 | 5.08E-21 | 0.72 | 1.00E+00 | 3.58E-01 | 1.66E-01 |
| Adenine | 0.34 | 1.00E+00 | 0.96 | 3.19E-01 | 0.29 | 1.00E+00 | 0.98 | 4.53E-01 | 0.98 | 1.00E+00 | 9.88E-01 | 3.02E-01 |
| Adipic_acid | 0.82 | 1.00E+00 | 0.92 | 1.58E-02 | 0.79 | 1.00E+00 | 1.1 | 4.34E-02 | 1.1 | 1.00E+00 | 8.56E-01 | 8.56E-03 |
| Alanine | 1.55 | 4.75E-04 | 0.87 | 2.83E-06 | 0.49 | 1.00E+00 | 0.96 | 2.09E-01 | 0.96 | 1.00E+00 | 8.49E-01 | 7.14E-01 |
| Allantoin | 1.36 | 1.19E-02 | 0.78 | 4.48E-05 | 0.06 | 1.00E+00 | 1.02 | 8.69E-01 | 1.02 | 1.00E+00 | 5.06E-01 | 9.00E-03 |
| Alpha-ketoglutaric_aicd | 1.68 | 1.33E-02 | 1.36 | 3.78E-07 | 0.95 | 7.93E-01 | 0.8 | 1.47E-02 | 0.8 | 1.00E+00 | 9.71E-01 | 6.00E-01 |
| Alpha-Linolenic_acid | 0.64 | 1.00E+00 | 1.16 | 5.95E-02 | 1.19 | 1.00E+00 | 1.24 | 2.08E-03 | 1.24 | 6.70E-01 | 2.25E-01 | 1.46E-01 |
| Aminoadipic_acid | 0.06 | 1.00E+00 | 1 | 8.51E-01 | 2.11 | 3.72E-05 | 0.63 | 2.24E-08 | 0.63 | 1.00E+00 | 2.49E-01 | 9.79E-01 |
| Anthranilate | 0.36 | 1.00E+00 | 0.96 | 2.88E-01 | 0.18 | 1.00E+00 | 1.01 | 6.41E-01 | 1.01 | 5.14E-01 | 2.18E-05 | 7.26E-01 |
| Arginine | 3.14 | 2.40E-22 | 0.61 | 1.35E-25 | 0.33 | 1.00E+00 | 1.05 | 3.99E-01 | 1.05 | 1.00E+00 | 8.56E-01 | 6.11E-01 |
| Asparagine | 0.51 | 1.00E+00 | 0.98 | 1.31E-01 | 0.15 | 1.00E+00 | 1.01 | 7.12E-01 | 1.01 | 1.00E+00 | 9.71E-01 | 2.14E-01 |
| Asparatic_acid | 3.51 | 7.85E-26 | 0.46 | 2.37E-34 | 0.87 | 1.00E+00 | 1.16 | 2.56E-02 | 1.16 | 1.00E+00 | 9.42E-01 | 7.35E-01 |
| Azelaic_acid | 0.8 |  | 0.79 | 1.79E-02 | 0.75 |  | 1.04 | 5.40E-02 | 1.04 |  |  |  |
| Benzoic_acid | 0.92 | 1.00E+00 | 0.91 | 6.65E-03 | 0.51 | 1.00E+00 | 1.07 | 1.93E-01 | 1.07 | 4.25E-01 | 4.25E-01 | 9.00E-03 |
| Biotin | 0.11 |  | 0.91 | 7.36E-01 | 0.26 |  | 0.87 | 5.09E-01 | 0.87 |  |  |  |
| Caffeine | 0.27 |  | 0.87 | 4.30E-01 | 0.61 |  | 0.94 | 1.21E-01 | 0.94 |  |  |  |
| Carnosine | 0.95 |  | 0.79 | 5.06E-03 | 0.85 |  | 1.18 | 2.86E-02 | 1.18 |  |  |  |
| Choline_chloride | 0.7 | 1.00E+00 | 0.9 | 3.84E-02 | 1.06 | 1.26E-01 | 1.17 | 6.31E-03 | 1.17 | 1.58E-01 | 1.58E-01 | 3.08E-02 |
| Citrulline | 0.69 | 1.00E+00 | 0.92 | 4.22E-02 | 0.36 | 1.00E+00 | 0.98 | 3.66E-01 | 0.98 | 1.00E+00 | 1.00E+00 | 2.18E-01 |
| Creatinine | 0.08 | 1.00E+00 | 1.01 | 8.04E-01 | 3.52 | 1.06E-20 | 0.74 | 4.36E-24 | 0.74 | 7.23E-02 | 7.23E-02 | 1.58E-03 |
| Cysteine | 0.08 | 1.00E+00 | 0.99 | 8.16E-01 | 0.02 | 1.00E+00 | 1 | 9.56E-01 | 1 | 1.00E+00 | 1.00E+00 | 5.46E-01 |
| Cytosine | 0.26 | 1.00E+00 | 1.02 | 4.40E-01 | 0.87 | 1.00E+00 | 1.09 | 2.53E-02 | 1.09 | 1.00E+00 | 1.00E+00 | 5.38E-01 |
| D-(-)-Arabinose | 0.38 | 1.00E+00 | 0.96 | 2.59E-01 | 0.47 | 1.00E+00 | 0.89 | 2.29E-01 | 0.89 | 1.00E+00 | 1.00E+00 | 6.84E-01 |
| D-glucuronic_acid | 0.89 | 1.00E+00 | 0.78 | 8.19E-03 | 1.76 | 9.04E-07 | 1.86 | 3.91E-06 | 1.86 | 6.31E-01 | 6.31E-01 | 4.54E-05 |
| D-Pantothenate | 0.04 | 1.00E+00 | 0.99 | 9.02E-01 | 0.63 | 1.00E+00 | 1.23 | 1.11E-01 | 1.23 | 1.00E+00 | 1.00E+00 | 9.03E-01 |
| dCMP | 0.47 |  | 0.74 | 1.64E-01 | 0.11 |  | 1.05 | 7.70E-01 | 1.05 |  |  |  |
| Dethiobiotin | 0.46 | 1.00E+00 | 0.99 | 1.76E-01 | 0.29 | 1.00E+00 | 0.98 | 4.54E-01 | 0.98 | 1.00E+00 | 1.00E+00 | 6.51E-01 |
| dGMP | 1 | 1.65E-01 | 0.84 | 3.03E-03 | 0.79 | 1.00E+00 | 1.11 | 4.20E-02 | 1.11 | 1.00E+00 | 1.00E+00 | 7.87E-01 |
| Dihydroorotate | 0.61 |  | 0.87 | 7.10E-02 | 0.16 |  | 1.02 | 6.91E-01 | 1.02 |  |  |  |
| Dimethyl_sulfone | 0.26 | 1.00E+00 | 0.9 | 4.53E-01 | 0.2 | 1.00E+00 | 0.97 | 6.10E-01 | 0.97 | 1.00E+00 | 1.00E+00 | 4.15E-01 |
| DL-2-Phenylpropionic_acid | 0.21 | 1.00E+00 | 0.97 | 5.43E-01 | 0.54 | 1.00E+00 | 1.11 | 1.74E-01 | 1.11 | 5.94E-01 | 5.94E-01 | 4.14E-02 |
| DL-Homocysteine | 0.91 | 1.00E+00 | 0.89 | 6.80E-03 | 0.55 | 1.00E+00 | 1.09 | 1.60E-01 | 1.09 | 1.00E+00 | 1.00E+00 | 4.14E-02 |
| DL-Kynurenine | 0.52 | 1.00E+00 | 0.93 | 1.26E-01 | 0.44 | 1.00E+00 | 0.97 | 2.67E-01 | 0.97 | 1.00E+00 | 1.00E+00 | 6.00E-01 |
| Dopamine | 2.39 | 7.33E-09 | 1.57 | 7.10E-14 | 0.31 | 1.00E+00 | 0.96 | 4.33E-01 | 0.96 | 5.78E-01 | 5.78E-01 | 7.91E-01 |
| Erythritol | 0.54 |  | 0.97 | 1.12E-01 | 0.29 |  | 0.85 | 4.63E-01 | 0.85 |  |  |  |
| Ethanolamine | 2.27 | 2.86E-09 | 1.27 | 1.54E-12 | 0.28 | 1.00E+00 | 1.03 | 4.72E-01 | 1.03 | 1.00E+00 | 1.00E+00 | 7.96E-01 |
| Ethyl_3-indoleacetate | 0.52 |  | 0.59 | 1.24E-01 | 0.63 |  | 1.49 | 1.10E-01 | 1.49 |  |  |  |
| Ethylene_glycol | 0.45 | 1.00E+00 | 0.96 | 1.85E-01 | 1.92 | 8.64E-06 | 1.19 | 4.03E-07 | 1.19 | 1.00E+00 | 1.00E+00 | 1.61E-02 |
| Fumarate | 0.28 | 1.00E+00 | 0.94 | 4.11E-01 | 0.33 | 1.00E+00 | 1.02 | 3.91E-01 | 1.02 | 1.00E+00 | 1.00E+00 | 7.61E-01 |
| Galactarate | 0.23 | 1.00E+00 | 1.01 | 5.05E-01 | 0.42 | 1.00E+00 | 1.02 | 2.78E-01 | 1.02 | 1.00E+00 | 1.00E+00 | 9.25E-01 |
| Gluconate | 0.44 |  | 0.93 | 1.97E-01 | 1.41 |  | 0.81 | 2.45E-04 | 0.81 |  |  |  |
| Gluconolactone | 0.12 | 1.00E+00 | 1.01 | 7.20E-01 | 0.39 | 1.00E+00 | 1.04 | 3.17E-01 | 1.04 | 1.00E+00 | 1.00E+00 | 7.26E-01 |
| Glucose | 1.75 | 9.10E-05 | 0.87 | 1.13E-07 | 0.45 | 1.00E+00 | 0.96 | 2.45E-01 | 0.96 | 1.00E+00 | 1.00E+00 | 6.24E-02 |
| Glutamic_acid | 0.31 | 1.00E+00 | 1.08 | 3.70E-01 | 0.3 | 1.00E+00 | 0.93 | 4.41E-01 | 0.93 | 1.00E+00 | 1.00E+00 | 6.51E-01 |
| Glutamine | 0.91 | 1.00E+00 | 0.92 | 7.14E-03 | 0.53 | 1.00E+00 | 1.05 | 1.76E-01 | 1.05 | 1.00E+00 | 1.00E+00 | 7.81E-02 |
| Glutaric_acid | 0.93 | 1.00E+00 | 0.91 | 5.90E-03 | 0.87 | 1.00E+00 | 1.1 | 2.59E-02 | 1.1 | 1.00E+00 | 1.00E+00 | 1.42E-02 |
| Glyceraldehyde | 0.11 | 1.00E+00 | 1 | 7.58E-01 | 0.68 | 1.00E+00 | 1.03 | 8.08E-02 | 1.03 | 1.00E+00 | 1.00E+00 | 3.38E-01 |
| Glyceraldehyde-3-phosphate | 1.39 | 3.67E-04 | 0.76 | 3.13E-05 | 0.1 | 1.00E+00 | 0.95 | 7.94E-01 | 0.95 | 1.00E+00 | 1.00E+00 | 9.25E-01 |
| Glycerol | 0.15 | 1.00E+00 | 0.99 | 6.66E-01 | 1.06 | 5.09E-01 | 1.07 | 6.06E-03 | 1.07 | 1.00E+00 | 1.00E+00 | 2.48E-02 |
| Glycine | 0.18 |  | 1.01 | 6.03E-01 | 0.38 |  | 1.06 | 3.25E-01 | 1.06 |  |  |  |
| GLycocyamine | 0.77 | 1.00E+00 | 0.92 | 2.38E-02 | 0.85 | 1.00E+00 | 1.1 | 3.00E-02 | 1.1 | 9.84E-02 | 9.84E-02 | 3.42E-04 |
| Glycolaldehyde_dimer | 1 | 1.00E+00 | 0.81 | 3.13E-03 | 0.03 | 1.00E+00 | 1.02 | 9.34E-01 | 1.02 | 1.00E+00 | 1.00E+00 | 4.71E-01 |
| Guanidinoacetate | 1.2 | 7.49E-02 | 0.94 | 3.42E-04 | 0.48 | 1.00E+00 | 1.02 | 2.22E-01 | 1.02 | 1.00E+00 | 1.00E+00 | 1.41E-01 |
| Guanine | 0.03 | 1.00E+00 | 1.03 | 9.21E-01 | 1.1 | 1.00E+00 | 1.25 | 4.38E-03 | 1.25 | 1.00E+00 | 1.00E+00 | 1.61E-02 |
| Hexanoic_acid | 0.7 | 1.00E+00 | 0.82 | 3.84E-02 | 2.42 | 1.42E-08 | 1.75 | 8.16E-11 | 1.75 | 5.62E-01 | 5.62E-01 | 2.24E-03 |
| Histidine | 1.14 | 3.37E-01 | 0.9 | 6.92E-04 | 1.47 | 1.72E-02 | 1.15 | 1.35E-04 | 1.15 | 5.90E-02 | 5.90E-02 | 9.00E-03 |
| Histidinol | 0.3 | 1.00E+00 | 0.99 | 3.84E-01 | 0.95 | 1.00E+00 | 1.04 | 1.45E-02 | 1.04 | 1.00E+00 | 1.00E+00 | 5.43E-01 |
| Homocysteine | 0.2 |  | 0.95 | 5.50E-01 | 0.02 |  | 0.97 | 9.60E-01 | 0.97 |  |  |  |
| Homogentisate | 0.08 | 1.00E+00 | 0.99 | 8.19E-01 | 0.25 | 1.00E+00 | 1.01 | 5.27E-01 | 1.01 | 1.00E+00 | 1.00E+00 | 4.48E-01 |
| Homovanillate | 0.84 | 1.00E+00 | 0.91 | 1.27E-02 | 0.75 | 1.00E+00 | 0.94 | 5.57E-02 | 0.94 | 1.00E+00 | 1.00E+00 | 5.43E-01 |
| Hydroquinone | 0.06 | 1.00E+00 | 0.99 | 8.49E-01 | 1.4 | 4.07E-02 | 1.08 | 2.70E-04 | 1.08 | 4.76E-01 | 4.76E-01 | 2.48E-02 |
| Hyoxyxanthine | 2.66 | 1.16E-10 | 1.65 | 1.94E-17 | 0.19 | 1.00E+00 | 0.97 | 6.30E-01 | 0.97 | 1.00E+00 | 1.00E+00 | 8.41E-01 |
| Indole-3-acetaldehyde | 0.92 | 1.00E+00 | 0.92 | 6.23E-03 | 0.2 | 1.00E+00 | 0.99 | 6.07E-01 | 0.99 | 1.00E+00 | 1.00E+00 | 3.88E-01 |
| Indole-3-acetic_acid | 2.93 | 1.49E-16 | 0.59 | 1.00E-21 | 0.94 | 3.43E-01 | 1.21 | 1.53E-02 | 1.21 | 1.00E+00 | 1.00E+00 | 2.14E-01 |
| Indoxyl_sulfate | 0.99 | 1.00E+00 | 0.91 | 3.16E-03 | 0.42 | 1.00E+00 | 0.97 | 2.79E-01 | 0.97 | 1.00E+00 | 1.00E+00 | 8.41E-01 |
| Isobutyric_acid | 0.71 | 1.00E+00 | 0.95 | 3.62E-02 | 0.98 | 5.33E-01 | 1.08 | 1.13E-02 | 1.08 | 1.00E+00 | 1.00E+00 | 3.43E-01 |
| Isocitric_acid | 0.24 | 1.00E+00 | 1.03 | 4.87E-01 | 0.26 | 1.00E+00 | 0.99 | 5.11E-01 | 0.99 | 1.00E+00 | 1.00E+00 | 3.67E-01 |
| Isovaleric_acid | 0.56 | 1.00E+00 | 0.94 | 9.75E-02 | 0.96 | 1.00E+00 | 0.91 | 1.34E-02 | 0.91 | 1.00E+00 | 1.00E+00 | 9.06E-01 |
| L-(-)-Glyceric_acid | 0.02 | 1.00E+00 | 0.96 | 9.56E-01 | 2.23 | 1.17E-06 | 1.54 | 2.89E-09 | 1.54 | 3.39E-01 | 3.39E-01 | 2.24E-03 |
| L-(-)-Pipecolinic_acid | 0.28 | 1.00E+00 | 0.95 | 4.11E-01 | 0.69 | 1.00E+00 | 1.28 | 7.86E-02 | 1.28 | 1.00E+00 | 1.00E+00 | 7.61E-01 |
| L-Homoserine | 0.39 | 1.00E+00 | 1.03 | 2.56E-01 | 1.8 | 9.84E-05 | 0.72 | 2.24E-06 | 0.72 | 1.00E+00 | 1.00E+00 | 3.38E-01 |
| L-Threonic_acid | 0.49 | 1.00E+00 | 0.89 | 1.53E-01 | 2.21 | 8.94E-07 | 1.4 | 4.38E-09 | 1.4 | 1.00E+00 | 1.00E+00 | 1.41E-01 |
| Lactate | 2.42 | 2.57E-11 | 1.42 | 2.50E-14 | 0.12 | 1.00E+00 | 1.03 | 7.58E-01 | 1.03 | 2.07E-02 | 2.07E-02 | 1.61E-02 |
| Lauroylcarnitine | 0.03 | 1.00E+00 | 0.95 | 9.41E-01 | 0.09 | 1.00E+00 | 1.04 | 8.23E-01 | 1.04 | 1.00E+00 | 1.00E+00 | 7.22E-01 |
| Leucine/Isoleucine | 0.83 | 1.00E+00 | 0.92 | 1.43E-02 | 0.43 | 1.00E+00 | 0.97 | 2.75E-01 | 0.97 | 1.00E+00 | 1.00E+00 | 3.47E-01 |
| Lysine | 0.88 | 1.00E+00 | 0.92 | 9.21E-03 | 0.53 | 1.00E+00 | 1.05 | 1.71E-01 | 1.05 | 1.00E+00 | 1.00E+00 | 7.81E-02 |
| Malate | 1.32 | 4.94E-03 | 1.24 | 8.13E-05 | 0.66 | 1.00E+00 | 0.91 | 9.08E-02 | 0.91 | 1.00E+00 | 1.00E+00 | 7.71E-01 |
| Maleic_acid | 0.49 | 1.00E+00 | 0.93 | 1.50E-01 | 1.65 | 2.46E-03 | 0.83 | 1.60E-05 | 0.83 | 1.00E+00 | 1.00E+00 | 4.32E-01 |
| Malonic_acid | 0.03 | 1.00E+00 | 1.01 | 9.34E-01 | 1.25 | 1.00E+00 | 1.68 | 1.26E-03 | 1.68 | 9.56E-01 | 9.56E-01 | 4.63E-01 |
| Mannitol | 2.44 | 7.32E-12 | 0.91 | 1.61E-14 | 0.1 | 1.00E+00 | 1 | 7.98E-01 | 1 | 1.00E+00 | 1.00E+00 | 8.50E-01 |
| meso-Erythritol | 0.86 | 1.00E+00 | 0.92 | 1.11E-02 | 0.45 | 1.00E+00 | 1.07 | 2.49E-01 | 1.07 | 2.43E-01 | 2.43E-01 | 2.56E-02 |
| Methionine | 0.33 | 1.00E+00 | 1.03 | 3.30E-01 | 0.25 | 1.00E+00 | 0.99 | 5.25E-01 | 0.99 | 8.55E-01 | 8.55E-01 | 2.92E-01 |
| Methyl_4-aminobutyrate | 0.77 | 1.00E+00 | 0.93 | 2.38E-02 | 0.5 | 1.00E+00 | 1.06 | 2.08E-01 | 1.06 | 1.00E+00 | 1.00E+00 | 2.05E-03 |
| Methyl_indole-3-acetate | 0.97 | 1.89E-01 | 0.9 | 3.93E-03 | 0.7 | 1.00E+00 | 1.05 | 7.05E-02 | 1.05 | 1.00E+00 | 1.00E+00 | 7.22E-01 |
| Methylmalonate | 0.86 | 1.00E+00 | 1.21 | 1.05E-02 | 0.06 | 1.00E+00 | 0.98 | 8.73E-01 | 0.98 | 1.00E+00 | 1.00E+00 | 7.86E-01 |
| Mevalolactone | 3.08 | 3.92E-22 | 0.61 | 1.90E-24 | 0.58 | 1.00E+00 | 1.08 | 1.39E-01 | 1.08 | 1.00E+00 | 1.00E+00 | 7.14E-01 |
| Mono-Methyl_Glutarate | 0.09 | 1.00E+00 | 0.98 | 7.85E-01 | 0.81 | 1.00E+00 | 1.07 | 3.61E-02 | 1.07 | 1.00E+00 | 1.00E+00 | 9.02E-01 |
| N-Acetyl-L-cysteine | 0.47 | 1.00E+00 | 0.98 | 1.70E-01 | 1.16 | 1.91E-01 | 1.06 | 2.86E-03 | 1.06 | 1.00E+00 | 1.00E+00 | 4.46E-02 |
| N-acetyl-L-leucine | 0.82 | 1.00E+00 | 0.96 | 1.49E-02 | 0.64 | 1.00E+00 | 1.02 | 1.00E-01 | 1.02 | 1.00E+00 | 1.00E+00 | 9.56E-01 |
| N-Acetylglycine | 0.69 | 1.00E+00 | 0.95 | 4.24E-02 | 0.51 | 1.00E+00 | 1.05 | 1.96E-01 | 1.05 | 1.00E+00 | 1.00E+00 | 6.00E-04 |
| N-acetylserotonin | 0.66 | 1.00E+00 | 1.16 | 5.01E-02 | 1.32 | 1.63E-02 | 0.73 | 6.42E-04 | 0.73 | 1.00E+00 | 1.00E+00 | 6.00E-01 |
| N,N-Dimethylglycine | 0.71 | 1.00E+00 | 0.9 | 3.67E-02 | 1.14 | 5.66E-02 | 1.18 | 3.19E-03 | 1.18 | 1.94E-01 | 1.94E-01 | 2.56E-02 |
| NADH | 0.73 |  | 0.58 | 3.14E-02 | 0.39 |  | 0.56 | 3.20E-01 | 0.56 |  |  |  |
| Noradrenaline | 0.75 | 1.00E+00 | 0.94 | 2.73E-02 | 1.98 | 1.14E-05 | 1.15 | 1.62E-07 | 1.15 | 1.00E+00 | 1.00E+00 | 1.13E-01 |
| O-Acetyl-L-carnitine | 0.43 | 1.00E+00 | 0.92 | 2.07E-01 | 1.28 | 3.06E-01 | 1.23 | 8.93E-04 | 1.23 | 1.00E+00 | 1.00E+00 | 4.66E-01 |
| O-methyl-D-serine | 0.37 | 1.00E+00 | 1.03 | 2.77E-01 | 1.83 | 8.44E-05 | 0.72 | 1.44E-06 | 0.72 | 1.00E+00 | 1.00E+00 | 3.38E-01 |
| Octopamine | 0.67 | 1.00E+00 | 0.96 | 4.88E-02 | 1.38 | 7.13E-02 | 1.07 | 3.55E-04 | 1.07 | 1.00E+00 | 1.00E+00 | 2.48E-01 |
| Ortho_Hydroxyphenylacetic_acid | 0.05 | 1.00E+00 | 0.95 | 8.86E-01 | 0.09 | 1.00E+00 | 1.02 | 8.12E-01 | 1.02 | 3.56E-02 | 3.56E-02 | 5.32E-02 |
| Oxaloacetic_acid | 0.51 |  | 0.92 | 1.33E-01 | 0.4 |  | 0.89 | 3.02E-01 | 0.89 |  |  |  |
| Paraxanthine | 0.19 |  | 0.92 | 5.79E-01 | 0.54 |  | 0.98 | 1.72E-01 | 0.98 |  |  |  |
| Pentanoate | 0.31 |  | 0.88 | 3.61E-01 | 0.7 |  | 0.87 | 7.18E-02 | 0.87 |  |  |  |
| Phenol | 0.84 | 1.00E+00 | 0.91 | 1.35E-02 | 0.08 | 1.00E+00 | 1.03 | 8.42E-01 | 1.03 | 1.00E+00 | 1.00E+00 | 3.95E-02 |
| PhenylAc-Gln-OH | 0.07 | 1.00E+00 | 0.97 | 8.48E-01 | 0.18 | 1.00E+00 | 1.01 | 6.50E-01 | 1.01 | 1.12E-01 | 1.12E-01 | 7.83E-01 |
| Phenylacetic_acid | 0.92 | 2.09E-01 | 1.28 | 6.55E-03 | 0.21 | 1.00E+00 | 0.96 | 5.91E-01 | 0.96 | 1.00E+00 | 1.00E+00 | 5.43E-01 |
| Phenylalanine | 3.29 | 4.26E-23 | 0.67 | 6.46E-29 | 0.5 | 1.00E+00 | 1.05 | 1.97E-01 | 1.05 | 1.00E+00 | 1.00E+00 | 6.13E-01 |
| Phospho(enol)_pyruvic_acid | 0.75 | 1.00E+00 | 0.95 | 2.58E-02 | 0.02 | 1.00E+00 | 1 | 9.51E-01 | 1 | 1.00E+00 | 1.00E+00 | 2.18E-01 |
| Phosphocholine | 1.12 | 3.41E-01 | 0.91 | 8.64E-04 | 0.97 | 1.00E+00 | 0.93 | 1.23E-02 | 0.93 | 1.00E+00 | 1.00E+00 | 8.80E-01 |
| Phosphoserine | 0.26 | 1.00E+00 | 0.91 | 4.53E-01 | 0.76 | 1.00E+00 | 1.18 | 5.23E-02 | 1.18 | 2.04E-01 | 2.04E-01 | 6.28E-03 |
| Piperine | 0.22 | 1.00E+00 | 0.92 | 5.20E-01 | 0.44 | 1.00E+00 | 1.28 | 2.55E-01 | 1.28 | 1.00E+00 | 1.00E+00 | 1.92E-01 |
| Proline | 0.46 | 1.00E+00 | 0.94 | 1.80E-01 | 1.95 | 1.30E-05 | 0.8 | 2.67E-07 | 0.8 | 1.00E+00 | 1.00E+00 | 7.92E-01 |
| PRPP | 0.64 | 1.00E+00 | 1.1 | 5.91E-02 | 1.01 | 1.00E+00 | 0.84 | 9.09E-03 | 0.84 | 1.00E+00 | 1.00E+00 | 3.88E-02 |
| Purine | 2.45 | 1.06E-12 | 0.68 | 1.05E-14 | 0.39 | 1.00E+00 | 1.05 | 3.16E-01 | 1.05 | 1.00E+00 | 1.00E+00 | 9.33E-01 |
| Pyridoxal | 0.53 |  | 0.73 | 1.20E-01 | 0.12 |  | 1.08 | 7.65E-01 | 1.08 |  |  |  |
| Pyruvate | 0.64 | 1.00E+00 | 0.95 | 5.97E-02 | 0.3 | 1.00E+00 | 1.01 | 4.49E-01 | 1.01 | 1.00E+00 | 1.00E+00 | 8.61E-02 |
| Pyruvic_aldehyde | 0.97 | 1.00E+00 | 0.91 | 4.11E-03 | 0.05 | 1.00E+00 | 1.02 | 9.02E-01 | 1.02 | 1.00E+00 | 1.00E+00 | 1.12E-01 |
| Quinate | 0.05 | 1.00E+00 | 0.95 | 8.78E-01 | 0.15 | 1.00E+00 | 0.94 | 7.07E-01 | 0.94 | 5.54E-01 | 5.54E-01 | 9.37E-01 |
| Resorcinol_monoacetate | 0.4 | 1.00E+00 | 0.98 | 2.43E-01 | 0.98 | 1.00E+00 | 1.04 | 1.15E-02 | 1.04 | 1.00E+00 | 1.00E+00 | 2.65E-01 |
| Ribitol | 0.54 | 1.00E+00 | 0.93 | 1.10E-01 | 1.21 | 3.27E-01 | 1.1 | 1.83E-03 | 1.1 | 1.00E+00 | 1.00E+00 | 1.29E-01 |
| S-Adenosyl-methionine | 0.44 |  | 1.15 | 1.96E-01 | 0.02 |  | 0.92 | 9.54E-01 | 0.92 |  |  |  |
| Salicylate | 0.59 | 1.00E+00 | 0.94 | 8.42E-02 | 1.21 | 5.30E-01 | 1.08 | 1.77E-03 | 1.08 | 1.00E+00 | 1.00E+00 | 2.84E-02 |
| Sarcosine | 0.99 | 4.87E-01 | 0.96 | 3.17E-03 | 0.51 | 1.00E+00 | 1.02 | 1.91E-01 | 1.02 | 1.00E+00 | 1.00E+00 | 1.41E-01 |
| Serine | 2.46 | 4.25E-11 | 0.73 | 8.39E-15 | 1.72 | 9.36E-04 | 1.22 | 6.49E-06 | 1.22 | 1.00E+00 | 1.00E+00 | 5.32E-02 |
| Serotonin | 1.55 | 6.74E-04 | 0.84 | 3.09E-06 | 1.03 | 1.00E+00 | 1.11 | 7.90E-03 | 1.11 | 1.00E+00 | 1.00E+00 | 5.80E-01 |
| Suberic_acid | 0.23 | 1.00E+00 | 1.02 | 4.90E-01 | 0.67 | 1.00E+00 | 1.04 | 8.36E-02 | 1.04 | 1.00E+00 | 1.00E+00 | 5.28E-02 |
| Succinic_acid | 0.25 |  | 1 | 4.71E-01 | 0.62 |  | 0.96 | 1.13E-01 | 0.96 |  |  |  |
| Sucrose | 0.24 | 1.00E+00 | 0.99 | 4.76E-01 | 0.64 | 1.00E+00 | 1.13 | 9.69E-02 | 1.13 | 1.00E+00 | 1.00E+00 | 5.59E-01 |
| Theobromine | 0.85 | 1.00E+00 | 0.85 | 1.15E-02 | 1.19 | 5.89E-02 | 1.24 | 2.02E-03 | 1.24 | 5.55E-02 | 5.55E-02 | 5.43E-03 |
| Theophylline | 0.24 | 1.00E+00 | 0.98 | 4.77E-01 | 0.55 | 1.00E+00 | 0.92 | 1.61E-01 | 0.92 | 1.00E+00 | 1.00E+00 | 7.14E-01 |
| Thiamine | 0.94 |  | 1.36 | 5.42E-03 | 0.29 |  | 0.99 | 4.52E-01 | 0.99 |  |  |  |
| Thiourea | 0.29 | 1.00E+00 | 0.97 | 3.90E-01 | 0.24 | 1.00E+00 | 1.03 | 5.39E-01 | 1.03 | 1.00E+00 | 1.00E+00 | 3.92E-01 |
| Threonine | 0.3 | 1.00E+00 | 0.97 | 3.80E-01 | 0.43 | 1.00E+00 | 1.05 | 2.75E-01 | 1.05 | 1.00E+00 | 1.00E+00 | 7.26E-01 |
| thymine | 2.85 | 4.71E-17 | 0.64 | 2.42E-20 | 0.6 | 1.00E+00 | 1.11 | 1.22E-01 | 1.11 | 1.00E+00 | 1.00E+00 | 3.56E-01 |
| Trans-Aconitic_acid | 0.19 | 1.00E+00 | 0.99 | 5.74E-01 | 1.51 | 5.46E-03 | 1.09 | 8.74E-05 | 1.09 | 3.25E-01 | 3.25E-01 | 2.82E-03 |
| Trans-Cinnamaldehyde | 1.08 | 1.30E-01 | 0.86 | 1.27E-03 | 1.22 | 2.79E-01 | 1.15 | 1.65E-03 | 1.15 | 3.77E-01 | 3.77E-01 | 4.59E-03 |
| Trigonelline | 0.15 | 1.00E+00 | 0.96 | 6.64E-01 | 0.1 | 1.00E+00 | 0.96 | 8.01E-01 | 0.96 | 2.64E-01 | 2.64E-01 | 8.85E-01 |
| Tryptophan | 0.63 | 1.00E+00 | 0.95 | 6.52E-02 | 0.05 | 1.00E+00 | 1 | 8.96E-01 | 1 | 1.00E+00 | 1.00E+00 | 1.61E-02 |
| Tyramine | 0.36 | 1.00E+00 | 0.95 | 2.90E-01 | 0.11 | 1.00E+00 | 0.98 | 7.72E-01 | 0.98 | 4.17E-01 | 4.17E-01 | 7.67E-01 |
| Tyrosine | 0.79 | 1.00E+00 | 0.92 | 1.94E-02 | 0.61 | 1.00E+00 | 0.95 | 1.15E-01 | 0.95 | 1.00E+00 | 1.00E+00 | 7.45E-01 |
| UDP-alpha-D-glucose | 0.58 | 1.00E+00 | 0.88 | 8.90E-02 | 0.45 | 1.00E+00 | 1.04 | 2.51E-01 | 1.04 | 6.51E-01 | 6.51E-01 | 7.72E-02 |
| Uracil | 0.65 | 1.00E+00 | 0.91 | 5.48E-02 | 0.08 | 1.00E+00 | 1 | 8.46E-01 | 1 | 1.00E+00 | 1.00E+00 | 7.05E-01 |
| Urate | 0.37 | 1.00E+00 | 0.97 | 2.76E-01 | 3 | 1.04E-14 | 0.83 | 8.39E-17 | 0.83 | 1.00E+00 | 1.00E+00 | 2.18E-01 |
| Uridine | 1.08 | 3.83E-01 | 1.19 | 1.29E-03 | 0.63 | 1.00E+00 | 0.92 | 1.10E-01 | 0.92 | 1.00E+00 | 1.00E+00 | 5.91E-01 |
| Urocanate | 0.15 | 1.00E+00 | 0.98 | 6.50E-01 | 0.28 | 1.00E+00 | 0.97 | 4.77E-01 | 0.97 | 1.00E+00 | 1.00E+00 | 8.13E-01 |
| Valine | 1.12 | 2.11E-01 | 0.9 | 8.77E-04 | 0.18 | 1.00E+00 | 0.99 | 6.50E-01 | 0.99 | 1.00E+00 | 1.00E+00 | 2.06E-01 |
| Xanthine | 0.61 | 1.00E+00 | 0.93 | 7.07E-02 | 0.09 | 1.00E+00 | 1.01 | 8.18E-01 | 1.01 | 1.00E+00 | 1.00E+00 | 8.85E-02 |
